# Priority Choice Experimental Two-Qubit Tomography: Measuring One by One All Elements of Density Matrices

**DOI:** 10.1038/srep19610

**Published:** 2016-01-21

**Authors:** Karol Bartkiewicz, Antonín Černoch, Karel Lemr, Adam Miranowicz

**Affiliations:** 1 Faculty of Physics, Adam Mickiewicz University, PL-61-614 Poznań, Poland; 2 RCPTM, Joint Laboratory of Optics of Palacký University and Institute of Physics of Academy of Sciences of the Czech Republic, 17. listopadu 12, 772 07 Olomouc, Czech Republic; 3 Institute of Physics of Academy of Sciences of the Czech Republic, Joint Laboratory of Optics of Palacký University and Institute of Physics of Academy of Sciences of the Czech Republic, 17. listopadu 50A, 77207 Olomouc, Czech Republic; 4 CEMS, RIKEN, 351-0198 Wako-shi, Japan

## Abstract

In standard optical tomographic methods, the off-diagonal elements of a density matrix *ρ* are measured indirectly. Thus, the reconstruction of *ρ*, even if it is based on linear inversion, typically magnifies small errors in the experimental data. Recently, an optimal tomography solution measuring all the elements of *ρ* one-by-one without error magnification has been theoretically proposed. We implemented this method for two-qubit polarization states. For comparison, we also experimentally implemented other well-known tomographic protocols, either based solely on local measurements (of, e.g., the Pauli operators and James-Kwiat-Munro-White projectors) or with mutually unbiased bases requiring both local and global measurements. We reconstructed seventeen separable, partially and maximally entangled two-qubit polarization states. Our experiments show that our method has the highest stability against errors in comparison to other quantum tomographies. In particular, we demonstrate that each optimally-reconstructed state is embedded in an uncertainty circle of the smallest radius, both in terms of trace distance and disturbance. We explain how to experimentally estimate uncertainty radii for all the implemented tomographies and show that, for each reconstructed state, the relevant uncertainty circles intersect indicating the approximate location of the corresponding physical density matrix.

Quantum tomographic methods are indispensable tools in experimental quantum physics. Indeed, characterizing quantum states and quantum processes are essential for studying the performance and evolution of quantum systems^1^ and for developing quantum technologies^2^. Both of these problems are mathematically equivalent, and are usually solved by applying quantum state tomography (QST). This approach is typically based on linear inversion^3^ and maximum-likelihood estimation^4^,^5^,^6^,^7^,^8^,^9^,^10^,^11^,^12^,^13^,^14^. There are also other approaches to quantum state estimation based on, e.g., least-squares inversion^15^, Bayesian mean estimation^1^,^16^,^17^, and linear regression estimation^18^. There exist dozens of QST protocols applicable even in the special case of photonic polarization state reconstruction (for a review see ref. 19 and also, e.g., refs 20, 21, 22, 23, 24, 25, 26, 27, 28). Thus, choosing the best protocol appeared to be a difficult task. However, a recent paper^29^ described an optimal QST protocol minimizing the condition number *κ* that characterizes the robustness against experimental errors. Condition numbers were also used for investigating the error stability of optical tomographic protocols in refs 21,30, 31, 32. In this Report we present an experimental study of this optimal protocol compared to four other popular approaches that use the same experimental setup (shown in Fig. 1) for the reconstruction of two polarization-entangled photons. The protocols in question include those based solely on the local measurements of (i) the James-Kwiat-Munro-White (JKMW) projectors^4^, (ii) the Pauli operators, and (iii) their eigenstates (the so-called standard basis)^20^,^33^, together with (iv) the protocol of Adamson and Steinberg^22^ based on mutually-unbiased bases (MUB) applying both local and global measurements, analogously to the optimal protocol. To compare these protocols we first derive a relation between the radius of the error circle associated with the reconstructed state and measured quantities. The radii correspond to the trace distance between the ideal density matrices and the reconstructed noisy ones. However, they can also be interpreted in terms of fidelity (or disturbance).

All the approaches analysed here are based on solving a linear-system problem *Ax* = *b*, where *A* is referred to as the *coefficient matrix*, *b* is the *observation vector* containing the measured data, and *x* = vec(*ρ*) is a real vector describing the unknown state *ρ* to be reconstructed. We choose


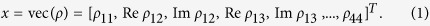


Conversely, the two-qubit density matrix *ρ* can be represented as a real vector *x* = (*x*_1_,..., *x*_16_) with its elements given as follows


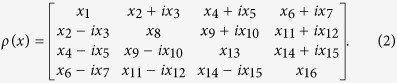


The already mentioned condition number *κ* depends only on *A*, i.e., the system of equations used to estimate the density matrix from the experimental data *b*. The reliability of the reconstructed density matrix *ρ*, which corresponds to the vector *x* = *A*^−1^*b* for a given set of rotations *A* (representing our linear tomographic system) and for measured data *b* depends on the value of *κ*. To show the operational (or physical) importance of condition numbers more explicitly, let us recall a well known theorem (Theorem 8.4 in ref. 34): Consider the system *Ax* = *b* with nonsingular *A*. Assume perturbations *δb* in *b*. If perturbations *δx* are defined implicitly by *A*(*x* + *δx*) = *b* + *δb*, then it holds^34^:


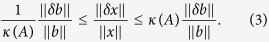


Thus, if the condition number *κ*(*A*), for a given norm, is equal (or very close) to one, then small relative changes in the observation vector *b* imply equally small relative changes in the reconstructed state *x*. Here we calculate


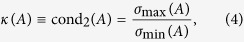


based on the spectral norm 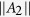, which is compatible with the Euclidean distance 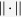 used for other quantities in Eq. (3). The norm is defined by the largest singular value of *A*, i.e, 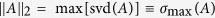, where the function svd (*A*) returns the singular values of *A*. As shown in ref. 29, optimal tomography provides *κ*(*A*) = 1 for 16 local and nonlocal measurements (composed of 28 projectors). By contrast, the JKMW tomography^4^ leads to 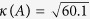 for 16 local measurements, the standard separable basis^20^,^33^ yields *κ*(*A*) = 3 for 36 local measurements, and the mutually-unbiased bases tomography^22^,^35^ gives 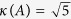 for 20 local and nonlocal measurements. The tomography based on Pauli matrices gives 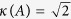 for 16 local measurements. This suggests that the density matrices reconstructed with these alternative protocols reside inside the uncertainty circles of various radii that depend on *κ*.

## Error robustness

In the Methods we define the uncertainty radius of the state estimation


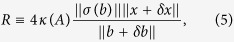


which we simply refer to as the maximum error being defined as the maximal trace distance between the state and its estimate, only in terms of the directly measured quantities, where *σ*(*b*) is a vector of the corresponding standard deviations (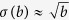 for the Poisson statistics). This is an important result, as it allows us to directly estimate the quality of a given state reconstruction in a very convenient way without knowing the state a priori. As we will demonstrate, it also makes it possible to visually compare the outcomes of various tomographies. As a result, it is easy to characterize the quality of reconstruction without knowing *ρ* a priori. Moreover, the uncertainty radius *R* can be used as a sanity check for the results of maximum likelihood methods, because proper density matrices should be contained within the uncertainty circle of this radius *R*.

We quantify the quality of a tomography protocol with the error *E* defined as the trace distance


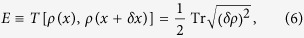


where *δρ* ≡ *ρ*(*δx*), between the ideal, *ρ*(*x*), and perturbed, *ρ*(*x* + *δx*), density matrices. Let us introduce 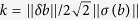, where 0 ≤ *k* ≤ 1 for the Poissonian statistics. In the most general case we can write


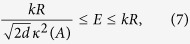


where the lower bound is derived with help of Eqs. (10) and (11), and the relation between the trace distance and the Hilbert-Schmidt distance 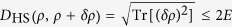. For an arbitrary distribution of the results *b*_*i*_, Chebyshev’s inequality implies that the probability of finding the reconstructed state inside of an error circle of the radius *kR* is bounded from below by 1 − 1/(8*k*^2^). This means that a minimum of 50% of values must lie within the 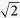 standard deviations of the mean regardless of the distribution, i.e., the value of *r* = *R*/2 bounds the median of the error *E* from above for any distribution. For the Poisson distribution, we can find a tighter upper bound on the probability of 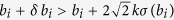 than the one provided by Chebyshev’s inequality, i.e., Pr(*X* > *x*) ≤ e^−*μ*^(e*μ*/*x*)^*x*^ (Theorem 5.4 in ref. 36), where *X* = *b*_*i*_ + *δb*_*i*_ is the random variable, *μ* = *b*_*i*_ and 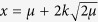.

## Experimental Results

**Figure 1:**
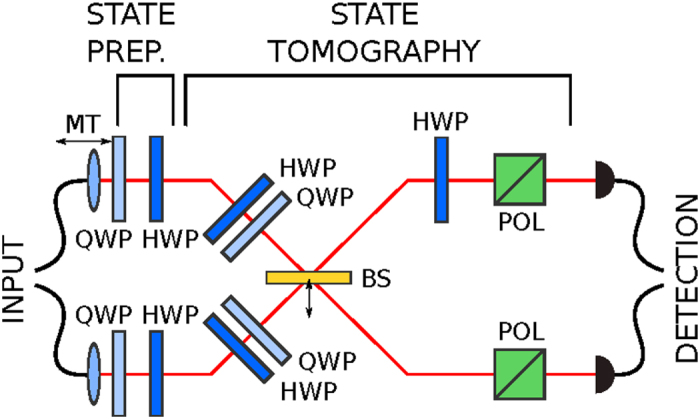
Experimental setup for performing both local and nonlocal polarization projections for the five studied tomographies. Linear-optical components are the quarter-wave plate (QWP), half-wave plate (HWP), horizontally retractable balanced beam splitter (BS), polarizing cube (POL), and motorized translation (MT) to stabilize the two-photon overlap.

**Figure 2:**
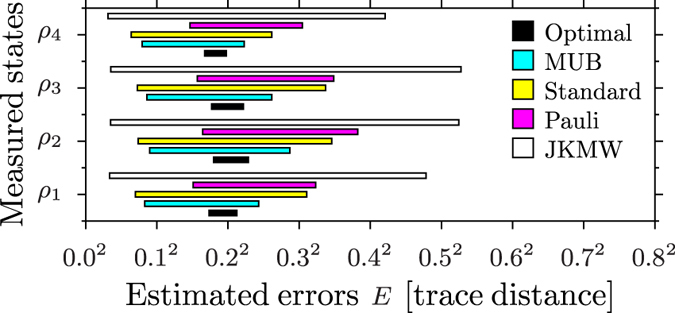
Experimentally recovered range of the most probable errors *E* for five tomographies (including the optimal, MUB, standard-separable-basis, Pauli operators, and JKMW protocols) for the four characteristic different two-qubit states approximated by 
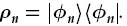 The shaded areas correspond to the most probable range of the error *E*, given by Eq. (7) for *k* = 1/2 (the upper and lower bounds on the plotted standard error) and the uncertainty radius *R*_||_, given by Eq. (13). The maximum error *R*_||_ is twice the upper limit *r* = *R*_||_/2 of the plotted error range. This error is known most precisely for the optimal tomography.

**Figure 3:**
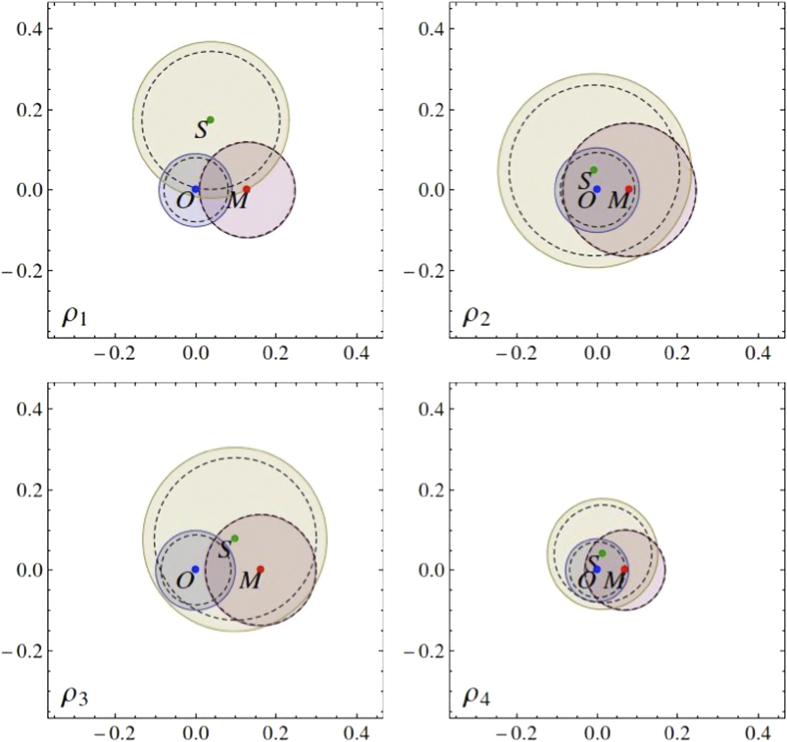
Relative trace distances between points corresponding to the states experimentally reconstructed by the optimal tomography (*O*), standard 36 state tomography (*S*), and MUB-based tomography (*M*) representing the reconstructed density matrices and their corresponding disks, with the radii *R*_||_ (solid circles) and *R* (dashed circles), describing the rescaled [*R*_||_ defined by Eq. (13)] and maximum errors [*R* defined by Eqs. (5) and (11)] for the four selected reconstructed states as in Fig. 2. All the graphically represented distances are scaled in the units of the trace distance. An ideally reconstructed state lies in the intersection of all the error disks with the radius *R*. Note that the discs with the radius *R*_||_/2, associated with the most probable range of errors *E* (see Fig. 2), do not necessarily intersect.

The results presented in the previous section suggest that the error *E* of state estimation depends both on the condition number *κ*(*A*) and the measured quantities *b*_*i*_. In order to compare the above-mentioned tomographic protocols using a single experimental setup shown in Fig. 1 we have prepared 17 two-qubit states of high purity and performed four tomographic protocols on each of them. One can find more details about the experimental setup in the Methods. The states are two-photon states described in the polarization basis {*HH*, *HV*, *VH*, *VV*}. This means that, e.g., *ρ*_11_ = *x*_1_ is the probability of detecting two photons in the polarization state 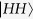 (both photons are polarized horizontally). The states range from maximally entangled to separable. Mixed states can be obtained by the weighed averaging of photon counts or density matrices associated with the 17 measured states (see the Supplement). However, here we present the results for the four most characteristics cases shown in Figs 2 and 3 as the other cases turn out to be qualitatively the same (see the Supplement). The four reconstructed states can be approximated with 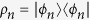, where 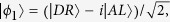 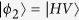, 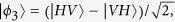 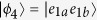, where 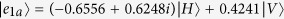 and 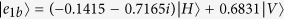 given in terms of the horizontal 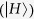, vertical 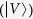, diagonal 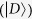, antidiagonal 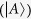, left-circular 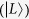, and right-circular 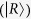 states.

The results of our analysis for our fair-comparison conditions (see the Methods) are shown in Fig. 2, which demonstrates that the error range *E* increases with the condition number *κ* in the same setup. This suggests that optimal tomography may indeed be the best solution. Even more convincing evidence is the analysis of the relative (trace) distances between the matrices reconstructed by various tomographies, and the sizes of their uncertainty circles *R*. We know that an unperturbed density matrix is found in the intersection of the error circles. Geometrically, this intersection is very close to the result of the optimal tomography because it has the smallest error radius. Four representative examples of this geometric construction are shown in Fig. 3. Our results for all the reconstructed states can be found in the Supplement.

## Conclusions

Our experiment is the first to have implemented the optimal two-qubit tomography and compare it with four other important tomographic protocols. This optimal method corresponds to measuring one by one all the elements *ρ*_*nm*_ of a density matrix *ρ*. This stands in contrast to the other protocols, where the off-diagonal elements of *ρ* are measured indirectly, i.e., the measured photon numbers correspond to linear combinations of some elements *ρ*_*nm*_. We have developed a method for estimating the error radii (in units of the trace distance) of circles containing the reconstructed density matrices. We have demonstrated that all the linear-inversion-based tomographies can be implemented and compared using the same framework. Our results confirm that the optimal tomography provides the most reliable results among all other analysed protocols. This makes the optimal tomography a method of choice, if the quality of the reconstructed density matrix is a priority. Another advantage of our method is that our setup uses only standard linear-optical elements and at the same time allows us to perform an arbitrary two-qubit state projections. Thus, it can be used for an arbitrary tomographic protocol. In our analysis we focused only on linear-inversion-based protocols where their error robustness could be quantified directly in a state-independent way without knowing the measured state in advance.

## Methods

### Experimental setup

We have generated separable and polarization-entangled photon pairs using the process of spontaneous parametric down-conversion occurring in a pair of BBO crystals (the so-called Kwiat *et al.* source^37^). For this source, we have observed about 2 × 10^3^ two-photon detections per second at 200 mW of pumping power at 355 nm. The generated photons were subsequently brought to the input of our tomography setup (see Fig. 1), where the required states were prepared. Next, we performed both the local and nonlocal polarizations projections. The preparation of a given state was achieved using pairs of half (HWP) and quarter (QWP) wave plates in the input mode of each photon.

In order to perform local projections on individual photons we have shifted the beam splitter BS horizontally so that the reflections were no longer coupled to the output ports. Then, for each local projection, we have adjusted the HWP and QWP in each photon’s path and then subjected the photons to the influence of a polarizing cube. Resulting two-photon detections were registered for 5 seconds. These projections are very accurate and the associated systematic error is much lower than the statistical one.

The nonlocal projections have been achieved by combining the local-state transformations using the HWPs and QWPs with the singlet-state projection on a balanced beam splitter (BS). For this, an additional HWP (set to 45°) has to be placed in one output mode of the beam splitter before the photons are subjected to the polarizers. Again, the two-photon detections were counted for 5 seconds. This procedure provides errors small in comparison to the statistical ones, as we make sure that the photons overlap as much as possible at the BS by finding its optimal position, while scanning the Hong-Ou-Mandel dip. Note that any small deviation in the BS parameters form the ideal 50:50 splitting ratio can be corrected during data postprocessing, since this deviation only affects the singlet-detection efficiency.

While the BS is clearly superfluous for local projections, and the polarizing cubes are unnecessary for the nonlocal projections, we had deliberately maintained all the components in the setup at all times, since we needed to compare the observed detection rates across local and nonlocal measurements. This would have been problematic without keeping all the components in the setup, since the components introduce different technological losses (e.g., back-reflections, scattering). Furthermore, our setup permits switching between local and nonlocal projections without much effort.

### Error estimation

From the linearity of the linear inversion problem and Eq. (3) it follows that


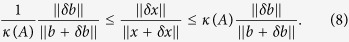


Let us quantify the quality of a tomography protocol with the trace distance given by Eq. (6), where *δρ* ≡ *ρ*(*δx*), between the ideal, *ρ*(*x*), and perturbed, *ρ*(*x* + *δx*), density matrices. The trace distance is a proper measure (metric) of the distance between two density matrices, and can be interpreted as a statistical measure of the probability of distinguishing between the two matrices. Moreover, it provides a single number that quantifies the error introduced by the protocol. We can relate *E* to 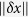 by using a standard inequality between the quadratic and arithmetic means of eigenvalues of 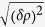. The result is


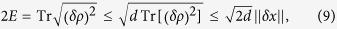


where, for a two-qubit density matrix, 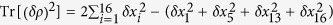 and the matrix dimension is *d* = 4. By combining inequalities in Eqs. (8) and (9) we arrive at


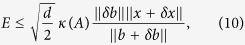


where the random deviations *δb* can be related to the vector of standard deviations, *σ*(*b*), associated with the mean values *b*. The distribution of random photon counts *b* + *δb* is usually described by the Poisson statistics. After performing the measurements *b* + *δb* one assumes that *b* + *δb* ≈ *b* = *σ*^2^(*b*), i.e., the measurement outcomes are the most probable (mean) number of counts. The relative error of this approximation is small if the number of counts is large. In order to compare the robustness of the tomographies, the deviations *δb* need to be bounded from above. For the Poisson distribution, the probability of a magnitude of a random deviation, 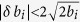, is given by its cumulative distribution function (CDF) as 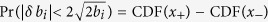, where 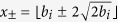 and for the Poisson distribution CDF(*x* < 0) = 0. The probability 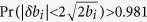 is very high for all *b*_*i*_. For *b*_*i*_ > 20 its value is already 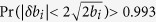. The same approach applied to the Gaussian distribution results in the widely used 3*σ* rule, which tells us that almost certainly (with probability 0.997) |*δb*_*i*_| < 3*σ*(*b*_*i*_). The statistically justified inequality 
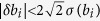
 leads to


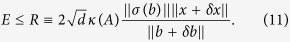


In order to characterize the quality of tomographic protocols we can also introduce the disturbance


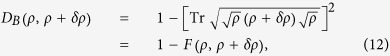


where *F* is the fidelity related to Bures metrics, which fulfils *D*_*B*_(*ρ*, *ρ* + *δρ*) ≤ *T*(*ρ*, *ρ* + *δρ*). Thus, *D*_*B*_ ≤ *E* ≤ *R*. This disturbance was used in ref. 22 for comparing the results of two-qubit tomographies. However, in our analysis we used trace distance instead of disturbance as it provided a more convenient theoretical framework.

For each tomography we have gathered the coincidence counts *b* + *δb* for specific projectors (see the Supplement). After performing the measurements we estimated the standard deviations as *b* + *δb* ≈ *b* = *σ*^2^(*b*). This is justified for large values of *b*_*i*_ as the relative error of estimating *σ*^2^(*b*_*i*_) from *b*_*i*_ + *δb*_*i*_ is 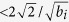. In our experiment, we have observed on average that *b*_*i*_ ≈ 10^3^ and the smallest values of *σ*(*b*_*i*_) do not contribute much to 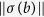. Thus, in order to correct for any possible underestimation of 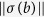, we rescale this value by a factor of 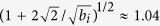. In most of the tomographies, the observation vector *b* + *δb* is measured directly. However, for the optimal tomography^29^ there are 12 measurements (out of 16) which correspond to the difference of two coincidence counts, say *c*_*i*_ and 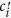. In these cases, the corresponding entries of the observation vector *b* are 
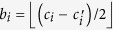
 are described by the Skellam distribution, where 
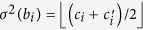
. We recall that the Skellam distribution is the discrete probability distribution of the difference between two statistically independent random variables each having the Poisson distributions with different mean values^38^. For the Skellam distribution (as for the Poisson distribution) the behaviour of the cumulative-distribution function implies that the largest disturbance can be limited (with probability >0.993 for 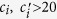) by 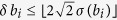.

### Fair comparison

In order to compare the protocols of different number of measurements, we additionally multiply *R* by a scaling factor 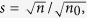 which fixes the total amount *n* of the state copies used in each measurement relative to the most efficient protocol which uses *n*_0_ copies. If the measurement are not performed in parallel, then *s* = 1 for all the protocols with 16 measurement, and 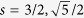 for the standard and MUB protocols, respectively. In this case, the optimal tomography is even better than it follows from our equal measurement time data described only by *R*. In the most experimentally demanding case of performing as many measurements in parallel as possible by using linear optics, the scaling factor reads 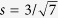 for all the analyzed protocols except for the MUB, where *s* = 1. In this case we denote the rescaled error radius


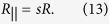


## Additional Information

**How to cite this article**: Bartkiewicz, K. *et al.* Priority Choice Experimental Two-Qubit Tomography: Measuring One by One All Elements of Density Matrices. *Sci. Rep.* **6**, 19610; doi: 10.1038/srep19610 (2016).

## Supplementary Material

Supplementary Information
