# Supplementary material for: Priority Choice Experimental Two-Qubit Tomography: Measuring One by One All Elements of Density Matrices
Source: Sci Rep. 2016 Jan 21;6:19610. doi: 10.1038/srep19610 (PMC4726363; doi:10.1038/srep19610)
Supplement: Supplementary Information [file srep19610-s1.pdf]

Priority Choice Experimental Two-qubit Tomography: Measuring One by One All Elements of Density Matrices: Supplementary Material

Karol Bartkiewicz,[1,2,*] Antonín Černoch,[3] Karel Lemr,[2] and Adam Miranowicz[4,1]

[1]*Faculty of Physics, Adam Mickiewicz University, PL-61-614 Poznań, Poland*
[2]*RCPTM, Joint Laboratory of Optics of Palacký University and Institute of Physics of Academy of Sciences of the Czech Republic, 17. listopadu 12, 772 07 Olomouc, Czech Republic*
[3]*Institute of Physics of Academy of Science of the Czech Republic, Joint Laboratory of Optics of Palacký University and Institute of Physics of Academy of Sciences of the Czech Republic, 17. listopadu 50A, 77207 Olomouc, Czech Republic*
[4]*CEMS, RIKEN, 351-0198 Wako-shi, Japan*

(Dated: October 22, 2015)

Here we show explicitly all the density matrices discussed in the Letter, which are reconstructed with the optimal tomographic protocol and those based on: (i) mutually unbiased bases, (ii) the James-Kwiat-Munro-White projectors, (iii) the tensor products of the Pauli operators, and (iv) the standard separable basis corresponding to all the eigenvectors of the Pauli operators. We also present the coefficient matrices, observation vectors corresponding to coincidence counts, the estimated variances for the observations, and the error radii for each reconstructed matrix. Finally, we compare the reconstructed matrices graphically, where we show the relative trace distances between the reconstructed states and they error radii.

* bark@amu.edu.pl

I. RECONSTRUCTED DENSITY MATRICES

The 17 density matrices are reconstructed by solving linear inversion problem for four tomographies. We have prepared 17 different states of high purity, which approximately correspond to:

$$\begin{aligned} |\psi_1\rangle &= (|HH\rangle - |VV\rangle)/\sqrt{2}, & |\psi_2\rangle &= (|HH\rangle + |VV\rangle)/\sqrt{2}, & |\psi_3\rangle &= (|HH\rangle - i|VV\rangle)/\sqrt{2}, \\ |\psi_4\rangle &= (|DR\rangle - i|AL\rangle)/\sqrt{2}, & |\psi_5\rangle &= (|HV\rangle + i|VH\rangle)/\sqrt{2}, & |\psi_6\rangle &= (|HV\rangle + |VH\rangle)/\sqrt{2}, \\ |\psi_7\rangle &= |HV\rangle, & |\psi_8\rangle &= (|HH\rangle + i|VV\rangle)/\sqrt{2}, & |\psi_9\rangle &= (|HV\rangle - |VH\rangle)/\sqrt{2}, \\ |\psi_{10}\rangle &= (|HV\rangle - i|VH\rangle)/\sqrt{2}, & |\psi_{11}\rangle &= (|DL\rangle + i|AR\rangle)/\sqrt{2}, & |\psi_{12}\rangle &= (|DL\rangle - i|AR\rangle)/\sqrt{2}, \\ |\psi_{13}\rangle &= |e_{1a}e_{1b}\rangle, & |\psi_{14}\rangle &= |e_{2a}e_{2b}\rangle, & |\psi_{15}\rangle &= 0.79|HV\rangle - 0.61|VH\rangle, \\ |\psi_{16}\rangle &= 0.50|HV\rangle - 0.87|VH\rangle, & |\psi_{17}\rangle &= 0.35|HV\rangle - 0.94|VH\rangle; \end{aligned} \quad (1)$$

where $|e_{1a}\rangle = (-0.6556 + 0.6248i)|H\rangle + 0.4241|V\rangle$, $|e_{1b}\rangle = (-0.1415 - 0.7165i)|H\rangle + 0.6831|V\rangle$, $|e_{2a}\rangle = (-0.9608 + 0.2091i)|H\rangle + 0.1822|V\rangle$, and $|e_{2b}\rangle = (0.2613 + 0.7338i)|H\rangle + 0.6271|V\rangle$ are the single photon-elliptic polarization states. In the subsequent sections, the states are numbered accordingly. Note that the states selected for the main text of our paper are defined as $|\phi_1\rangle \equiv |\psi_4\rangle$, $|\phi_2\rangle \equiv |\psi_7\rangle$, $|\phi_3\rangle \equiv |\psi_9\rangle$, and $|\phi_4\rangle \equiv |\psi_{14}\rangle$. We mark the data relevant to a particular tomography as follows: index O for the optimal tomography; S for the standard 36-state tomography; J for the James-Kwiat-Munro-White (JKMW) protocol; M for the MUB-based tomography; P for the Pauli-matrices-based tomography.

A. Standard 36-state tomography

$$\rho_{S,1} = \begin{bmatrix} 0.4922 & 0.0020+0.0156i & -0.0042+0.0354i & -0.4607-0.0750i \\ 0.0020-0.0156i & 0.0047 & -0.0054+0.0228i & 0.0255-0.0002i \\ -0.0042-0.0354i & -0.0054-0.0228i & 0.0136 & 0.0184+0.0656i \\ -0.4607+0.0750i & 0.0255+0.0002i & 0.0184-0.0656i & 0.4895 \end{bmatrix}$$

$$\rho_{S,2} = \begin{bmatrix} 0.4870 & 0.0029-0.0358i & 0.0237-0.0103i & 0.4723+0.0515i \\ 0.0029+0.0358i & 0.0085 & -0.0000-0.0219i & 0.0244+0.0413i \\ 0.0237+0.0103i & -0.0000+0.0219i & 0.0038 & -0.0052-0.0378i \\ 0.4723-0.0515i & 0.0244-0.0413i & -0.0052+0.0378i & 0.5007 \end{bmatrix}$$

$$\rho_{S,3} = \begin{bmatrix} 0.5363 & 0.0744-0.0611i & 0.0830-0.0243i & -0.0027+0.4636i \\ 0.0744+0.0611i & 0.0206 & 0.0522-0.0036i & -0.0513+0.0602i \\ 0.0830+0.0243i & 0.0522+0.0036i & 0.0002 & 0.0005+0.0402i \\ -0.0027-0.4636i & -0.0513-0.0602i & 0.0005-0.0402i & 0.4429 \end{bmatrix}$$

$$\rho_{S,4} = \begin{bmatrix} 0.3005 & 0.2633+0.0250i & 0.0456-0.2223i & -0.0648+0.2710i \\ 0.2633-0.0250i & 0.2482 & 0.0417-0.1841i & -0.0334+0.2402i \\ 0.0456+0.2223i & 0.0417+0.1841i & 0.1270 & -0.2145-0.0463i \\ -0.0648-0.2710i & -0.0334-0.2402i & -0.2145+0.0463i & 0.3244 \end{bmatrix}$$

$$\rho_{S,5} = \begin{bmatrix} 0.0135 & -0.0859+0.0013i & 0.0334+0.0685i & 0.0159+0.0018i \\ -0.0859-0.0013i & 0.5111 & 0.0397-0.4647i & -0.0002-0.0441i \\ 0.0334-0.0685i & 0.0397+0.4647i & 0.4697 & 0.0078-0.0236i \\ 0.0159-0.0018i & -0.0002+0.0441i & 0.0078+0.0236i & 0.0056 \end{bmatrix}$$

$$\rho_{S,6} = \begin{bmatrix} 0.0146 & 0.0450+0.0866i & 0.0849+0.0819i & -0.0046+0.0341i \\ 0.0450-0.0866i & 0.4606 & 0.4608-0.0160i & -0.0222-0.0282i \\ 0.0849-0.0819i & 0.4608+0.0160i & 0.5177 & -0.0526-0.0125i \\ -0.0046-0.0341i & -0.0222+0.0282i & -0.0526+0.0125i & 0.0072 \end{bmatrix}$$

$$\rho_{S,7} = \begin{bmatrix} 0.0005 & 0.0578-0.0482i & 0.0064+0.0028i & -0.0088-0.0005i \\ 0.0578+0.0482i & 0.9915 & 0.0290+0.0678i & -0.0436-0.1064i \\ 0.0064-0.0028i & 0.0290-0.0678i & 0.0049 & -0.0043-0.0022i \\ -0.0088+0.0005i & -0.0436+0.1064i & -0.0043+0.0022i & 0.0032 \end{bmatrix}$$

$$\rho_{S,8} = \begin{bmatrix} 0.5609 & -0.0543+0.0315i & 0.0357-0.0364i & 0.0027-0.4704i \\ -0.0543-0.0315i & 0.0067 & -0.0470+0.0079i & -0.0016+0.0511i \\ 0.0357+0.0364i & -0.0470-0.0079i & -0.0091 & 0.0065-0.0126i \\ 0.0027+0.4704i & -0.0016-0.0511i & 0.0065+0.0126i & 0.4416 \end{bmatrix}$$

$$\rho_{S,9} = \begin{bmatrix} -0.0208 & -0.0024-0.0670i & -0.0036+0.0319i & -0.0224-0.0403i \\ -0.0024+0.0670i & 0.5767 & -0.4584-0.0718i & -0.0076-0.0478i \\ -0.0036-0.0319i & -0.4584+0.0718i & 0.4334 & 0.0134+0.0045i \\ -0.0224+0.0403i & -0.0076+0.0478i & 0.0134-0.0045i & 0.0107 \end{bmatrix}$$

$$\rho_{S,10} = \begin{bmatrix} -0.0118 & 0.0243-0.0103i & 0.0134+0.0063i & -0.0090-0.0064i \\ 0.0243+0.0103i & 0.5080 & 0.0499+0.4684i & -0.0174-0.0050i \\ 0.0134-0.0063i & 0.0499-0.4684i & 0.4801 & 0.0302+0.0537i \\ -0.0090+0.0064i & -0.0174+0.0050i & 0.0302-0.0537i & 0.0237 \end{bmatrix}$$

$$\rho_{S,11} = \begin{bmatrix} 0.2826 & 0.2502+0.0153i & -0.0157+0.2358i & 0.0053-0.2593i \\ 0.2502-0.0153i & 0.2221 & -0.0295+0.2241i & 0.0222-0.2349i \\ -0.0157-0.2358i & -0.0295-0.2241i & 0.2718 & -0.2432+0.0003i \\ 0.0053+0.2593i & 0.0222+0.2349i & -0.2432-0.0003i & 0.2235 \end{bmatrix}$$

$$\rho_{S,12} = \begin{bmatrix} 0.2100 & -0.2611-0.0385i & -0.0419-0.2516i & 0.0345-0.2156i \\ -0.2611+0.0385i & 0.2815 & 0.0452+0.2460i & 0.0115+0.2405i \\ -0.0419+0.2516i & 0.0452-0.2460i & 0.2312 & 0.2259+0.0926i \\ 0.0345+0.2156i & 0.0115-0.2405i & 0.2259-0.0926i & 0.2772 \end{bmatrix}$$

$$\rho_{S,13} = \begin{bmatrix} 0.2849 & -0.2477-0.0400i & 0.0073+0.2296i & 0.0167+0.2654i \\ -0.2477+0.0400i & 0.2042 & 0.0104-0.2183i & -0.0427-0.2274i \\ 0.0073-0.2296i & 0.0104+0.2183i & 0.2687 & 0.2297-0.0240i \\ 0.0167-0.2654i & -0.0427+0.2274i & 0.2297+0.0240i & 0.2422 \end{bmatrix}$$

$$\rho_{S,14} = \begin{bmatrix} 0.3786 & -0.0779-0.3764i & -0.1289+0.1049i & 0.1243+0.0775i \\ -0.0779+0.3764i & 0.3745 & -0.0776-0.1422i & -0.0950+0.1000i \\ -0.1289-0.1049i & -0.0776+0.1422i & 0.1345 & -0.0377-0.1163i \\ 0.1243-0.0775i & -0.0950-0.1000i & -0.0377+0.1163i & 0.1124 \end{bmatrix}$$

$$\rho_{S,15} = \begin{bmatrix} 0.5241 & 0.1701+0.4098i & -0.0732+0.0497i & -0.0613-0.0740i \\ 0.1701-0.4098i & 0.3733 & 0.0048+0.0800i & -0.0776+0.0176i \\ -0.0732-0.0497i & 0.0048-0.0800i & 0.0586 & 0.0165+0.0519i \\ -0.0613+0.0740i & -0.0776-0.0176i & 0.0165-0.0519i & 0.0440 \end{bmatrix}$$

$$\rho_{S,16} = \begin{bmatrix} 0.0079 & -0.0088-0.1191i & -0.0026+0.0469i & -0.0179-0.0369i \\ -0.0088+0.1191i & 0.6443 & -0.4307-0.0597i & -0.0144-0.0826i \\ -0.0026-0.0469i & -0.4307+0.0597i & 0.3254 & 0.0248+0.0298i \\ -0.0179+0.0369i & -0.0144+0.0826i & 0.0248-0.0298i & 0.0224 \end{bmatrix}$$

$$\rho_{S,17} = \begin{bmatrix} 0.0071 & -0.0098-0.1224i & -0.0079+0.0450i & -0.0180-0.0331i \\ -0.0098+0.1224i & 0.7482 & -0.3769-0.0542i & -0.0215-0.1008i \\ -0.0079-0.0450i & -0.3769+0.0542i & 0.2232 & 0.0274+0.0197i \\ -0.0180+0.0331i & -0.0215+0.1008i & 0.0274-0.0197i & 0.0215 \end{bmatrix}$$

B. JKMW Tomography

$$\rho_{J,1} = \begin{bmatrix} 0.4879 & -0.0241+0.0194i & -0.0198+0.0473i & -0.4503-0.0438i \\ -0.0241-0.0194i & 0.0054 & -0.0313+0.1193i & 0.0428-0.0066i \\ -0.0198-0.0473i & -0.0313-0.1193i & 0.0225 & -0.0023+0.0852i \\ -0.4503+0.0438i & 0.0428+0.0066i & -0.0023-0.0852i & 0.4842 \end{bmatrix}$$

$$\rho_{J,2} = \begin{bmatrix} 0.4748 & 0.0156-0.0702i & -0.0009+0.0089i & 0.4543+0.0190i \\ 0.0156+0.0702i & 0.0107 & 0.0457-0.1052i & 0.0248+0.0104i \\ -0.0009-0.0089i & 0.0457+0.1052i & 0.0079 & 0.0098-0.0398i \\ 0.4543-0.0190i & 0.0248-0.0104i & 0.0098+0.0398i & 0.5065 \end{bmatrix}$$

$$\rho_{J,3} = \begin{bmatrix} 0.5221 & 0.0753-0.0707i & 0.0706-0.0238i & 0.0314+0.4451i \\ 0.0753+0.0707i & 0.0213 & 0.0016-0.0338i & -0.0532+0.0446i \\ 0.0706+0.0238i & 0.0016+0.0338i & 0.0112 & 0.0008+0.0618i \\ 0.0314-0.4451i & -0.0532-0.0446i & 0.0008-0.0618i & 0.4454 \end{bmatrix}$$

$$\rho_{J,4} = \begin{bmatrix} 0.2807 & 0.2535+0.0302i & 0.0392-0.1853i & -0.0417+0.2515i \\ 0.2535-0.0302i & 0.2543 & -0.0142-0.1473i & -0.0489+0.1999i \\ 0.0392+0.1853i & -0.0142+0.1473i & 0.1256 & -0.2106-0.0075i \\ -0.0417-0.2515i & -0.0489-0.1999i & -0.2106+0.0075i & 0.3394 \end{bmatrix}$$

$$\rho_{J,5} = \begin{bmatrix} 0.0260 & -0.0955+0.0352i & 0.0370+0.0450i & 0.0842+0.0143i \\ -0.0955-0.0352i & 0.5035 & 0.0139-0.4182i & -0.0177-0.0134i \\ 0.0370-0.0450i & 0.0139+0.4182i & 0.4676 & -0.0199-0.0215i \\ 0.0842-0.0143i & -0.0177+0.0134i & -0.0199+0.0215i & 0.0029 \end{bmatrix}$$

$$\rho_{J,6} = \begin{bmatrix} 0.0367 & 0.0389+0.1059i & 0.0675+0.0395i & 0.0312+0.0146i \\ 0.0389-0.1059i & 0.4500 & 0.4370-0.0716i & -0.0533-0.0099i \\ 0.0675-0.0395i & 0.4370+0.0716i & 0.5064 & -0.0482-0.0141i \\ 0.0312-0.0146i & -0.0533+0.0099i & -0.0482+0.0141i & 0.0069 \end{bmatrix}$$

$$\rho_{J,7} = \begin{bmatrix} 0.0062 & 0.0456-0.0314i & 0.0057+0.0030i & 0.0072-0.0079i \\ 0.0456+0.0314i & 0.9818 & 0.0226+0.0910i & -0.0969-0.0714i \\ 0.0057-0.0030i & 0.0226-0.0910i & 0.0032 & -0.0035+0.0032i \\ 0.0072+0.0079i & -0.0969+0.0714i & -0.0035-0.0032i & 0.0087 \end{bmatrix}$$

$$\rho_{J,8} = \begin{bmatrix} 0.5550 & -0.0784+0.0200i & -0.0058-0.0372i & -0.0223-0.4409i \\ -0.0784-0.0200i & 0.0135 & 0.0219+0.0377i & -0.0032+0.0374i \\ -0.0058+0.0372i & 0.0219-0.0377i & 0.0043 & -0.0027-0.0162i \\ -0.0223+0.4409i & -0.0032-0.0374i & -0.0027+0.0162i & 0.4271 \end{bmatrix}$$

$$\rho_{J,9} = \begin{bmatrix} 0.0089 & -0.0324-0.0301i & -0.0031-0.0189i & 0.0009-0.0043i \\ -0.0324+0.0301i & 0.5684 & -0.4348+0.0409i & -0.0166-0.0446i \\ -0.0031+0.0189i & -0.4348-0.0409i & 0.4209 & 0.0002-0.0151i \\ 0.0009+0.0043i & -0.0166+0.0446i & 0.0002+0.0151i & 0.0018 \end{bmatrix}$$

$$\rho_{J,10} = \begin{bmatrix} 0.0058 & 0.0400-0.0019i & 0.0128-0.0240i & -0.0401-0.0454i \\ 0.0400+0.0019i & 0.5133 & 0.0953+0.3781i & -0.0293+0.0144i \\ 0.0128+0.0240i & 0.0953-0.3781i & 0.4753 & 0.0619+0.0094i \\ -0.0401+0.0454i & -0.0293-0.0144i & 0.0619-0.0094i & 0.0056 \end{bmatrix}$$

$$\rho_{J,11} = \begin{bmatrix} 0.2918 & 0.2453-0.0200i & -0.0285+0.1767i & -0.0527-0.2169i \\ 0.2453+0.0200i & 0.1992 & 0.0202+0.2358i & 0.0220-0.2014i \\ -0.0285-0.1767i & 0.0202-0.2358i & 0.2948 & -0.2464+0.0008i \\ -0.0527+0.2169i & 0.0220+0.2014i & -0.2464-0.0008i & 0.2142 \end{bmatrix}$$

$$\rho_{J,12} = \begin{bmatrix} 0.1923 & -0.2367-0.0390i & -0.0338-0.1873i & -0.0453-0.2798i \\ -0.2367+0.0390i & 0.3002 & 0.0996+0.1912i & 0.0039+0.2219i \\ -0.0338+0.1873i & 0.0996-0.1912i & 0.2127 & 0.2404+0.0740i \\ -0.0453+0.2798i & 0.0039-0.2219i & 0.2404-0.0740i & 0.2947 \end{bmatrix}$$

$$\rho_{J,13} = \begin{bmatrix} 0.3064 & -0.2350-0.0157i & 0.0162+0.1740i & 0.0769+0.2162i \\ -0.2350+0.0157i & 0.1884 & -0.0753-0.2582i & -0.0327-0.1833i \\ 0.0162-0.1740i & -0.0753+0.2582i & 0.2840 & 0.2538-0.0131i \\ 0.0769-0.2162i & -0.0327+0.1833i & 0.2538+0.0131i & 0.2211 \end{bmatrix}$$

$$\rho_{J,14} = \begin{bmatrix} 0.3803 & -0.0676-0.4027i & -0.1333+0.1142i & 0.1197+0.1053i \\ -0.0676+0.4027i & 0.3748 & -0.0745-0.1464i & -0.0932+0.1079i \\ -0.1333-0.1142i & -0.0745+0.1464i & 0.1345 & -0.0335-0.1211i \\ 0.1197-0.1053i & -0.0932-0.1079i & -0.0335+0.1211i & 0.1104 \end{bmatrix}$$

$$\rho_{J,15} = \begin{bmatrix} 0.5276 & 0.1690+0.4181i & -0.0627+0.0920i & -0.0903-0.0653i \\ 0.1690-0.4181i & 0.3819 & 0.0342+0.0807i & -0.0764+0.0379i \\ -0.0627-0.0920i & 0.0342-0.0807i & 0.0478 & 0.0032+0.0440i \\ -0.0903+0.0653i & -0.0764-0.0379i & 0.0032-0.0440i & 0.0427 \end{bmatrix}$$

$$\rho_{J,16} = \begin{bmatrix} 0.0207 & -0.0045-0.1146i & -0.0112+0.0073i & -0.0325-0.0020i \\ -0.0045+0.1146i & 0.6312 & -0.4297+0.0089i & 0.0034-0.0464i \\ -0.0112-0.0073i & -0.4297-0.0089i & 0.3450 & 0.0040+0.0183i \\ -0.0325+0.0020i & 0.0034+0.0464i & 0.0040-0.0183i & 0.0031 \end{bmatrix}$$

$$\rho_{J,17} = \begin{bmatrix} 0.0202 & -0.0172-0.1220i & -0.0095+0.0268i & -0.0629-0.0175i \\ -0.0172+0.1220i & 0.7415 & -0.3322-0.0029i & -0.0138-0.0503i \\ -0.0095-0.0268i & -0.3322+0.0029i & 0.2341 & 0.0108-0.0009i \\ -0.0629+0.0175i & -0.0138+0.0503i & 0.0108+0.0009i & 0.0042 \end{bmatrix}$$

C. MUB-based tomography

$$\rho_{M,1} = \begin{bmatrix} 0.4789 & 0.0857+0.0275i & 0.0044+0.0534i & -0.4699-0.0476i \\ 0.0857-0.0275i & 0.0311 & -0.0110-0.0029i & 0.0332-0.0196i \\ 0.0044-0.0534i & -0.0110+0.0029i & -0.0015 & 0.0215+0.0758i \\ -0.4699+0.0476i & 0.0332+0.0196i & 0.0215-0.0758i & 0.4915 \end{bmatrix}$$

$$\rho_{M,2} = \begin{bmatrix} 0.4888 & -0.0083-0.0456i & 0.0198+0.0013i & 0.4919+0.0541i \\ -0.0083+0.0456i & 0.0296 & 0.0027-0.0241i & 0.0206+0.0291i \\ 0.0198-0.0013i & 0.0027+0.0241i & -0.0322 & -0.0820-0.0477i \\ 0.4919-0.0541i & 0.0206-0.0291i & -0.0820+0.0477i & 0.5138 \end{bmatrix}$$

$$\rho_{M,3} = \begin{bmatrix} 0.5214 & 0.0980 - 0.0452i & 0.0865 - 0.0535i & 0.0541 + 0.4566i \\ 0.0980 + 0.0452i & 0.0573 & -0.0021 + 0.0016i & -0.0472 + 0.0870i \\ 0.0865 + 0.0535i & -0.0021 - 0.0016i & 0.0053 & -0.0563 + 0.0557i \\ 0.0541 - 0.4566i & -0.0472 - 0.0870i & -0.0563 - 0.0557i & 0.4160 \end{bmatrix}$$

$$\rho_{M,4} = \begin{bmatrix} 0.2423 & 0.2560 + 0.0449i & 0.0503 - 0.2014i & 0.0328 + 0.2533i \\ 0.2560 - 0.0449i & 0.3304 & 0.1157 - 0.1609i & -0.0336 + 0.2319i \\ 0.0503 + 0.2014i & 0.1157 + 0.1609i & 0.1676 & -0.1754 - 0.0308i \\ 0.0328 - 0.2533i & -0.0336 - 0.2319i & -0.1754 + 0.0308i & 0.2597 \end{bmatrix}$$

$$\rho_{M,5} = \begin{bmatrix} -0.0004 & -0.0941 + 0.0188i & 0.0384 + 0.0899i & 0.0017 - 0.0313i \\ -0.0941 - 0.0188i & 0.4975 & 0.1830 - 0.4264i & 0.0052 - 0.0638i \\ 0.0384 - 0.0899i & 0.1830 + 0.4264i & 0.5160 & 0.0302 - 0.0058i \\ 0.0017 + 0.0313i & 0.0052 + 0.0638i & 0.0302 + 0.0058i & -0.0131 \end{bmatrix}$$

$$\rho_{M,6} = \begin{bmatrix} 0.0119 & 0.0116 + 0.0964i & 0.0767 + 0.0744i & 0.0106 + 0.0150i \\ 0.0116 - 0.0964i & 0.4477 & 0.4659 + 0.0031i & -0.0301 - 0.0330i \\ 0.0767 - 0.0744i & 0.4659 - 0.0031i & 0.5525 & -0.0780 - 0.0024i \\ 0.0106 - 0.0150i & -0.0301 + 0.0330i & -0.0780 + 0.0024i & -0.0121 \end{bmatrix}$$

$$\rho_{M,7} = \begin{bmatrix} 0.0377 & 0.0294 - 0.0443i & 0.0050 - 0.0081i & -0.0075 + 0.0315i \\ 0.0294 + 0.0443i & 0.9628 & 0.0006 + 0.0373i & -0.0462 - 0.1064i \\ 0.0050 + 0.0081i & 0.0006 - 0.0373i & 0.0047 & 0.0296 + 0.0029i \\ -0.0075 - 0.0315i & -0.0462 + 0.1064i & 0.0296 - 0.0029i & -0.0052 \end{bmatrix}$$

$$\rho_{M,8} = \begin{bmatrix} 0.5549 & -0.0058 + 0.0214i & 0.0324 + 0.0106i & -0.0598 - 0.4494i \\ -0.0058 - 0.0214i & 0.0236 & 0.0055 - 0.0171i & -0.0052 + 0.0074i \\ 0.0324 - 0.0106i & 0.0055 + 0.0171i & -0.0329 & -0.0199 - 0.0231i \\ -0.0598 + 0.4494i & -0.0052 - 0.0074i & -0.0199 + 0.0231i & 0.4544 \end{bmatrix}$$

$$\rho_{M,9} = \begin{bmatrix} -0.0157 & -0.0020 - 0.0764i & -0.0028 + 0.0180i & -0.0136 - 0.0240i \\ -0.0020 + 0.0764i & 0.5405 & -0.4696 - 0.0885i & -0.0067 - 0.0289i \\ -0.0028 - 0.0180i & -0.4696 + 0.0885i & 0.4525 & 0.0798 - 0.0047i \\ -0.0136 + 0.0240i & -0.0067 + 0.0289i & 0.0798 + 0.0047i & 0.0228 \end{bmatrix}$$

$$\rho_{M,10} = \begin{bmatrix} -0.0061 & -0.0415 - 0.0233i & 0.0029 - 0.0435i & -0.0009 - 0.0034i \\ -0.0415 + 0.0233i & 0.4724 & -0.0159 + 0.4682i & -0.0280 + 0.0304i \\ 0.0029 + 0.0435i & -0.0159 - 0.4682i & 0.4984 & 0.0302 + 0.0410i \\ -0.0009 + 0.0034i & -0.0280 - 0.0304i & 0.0302 - 0.0410i & 0.0353 \end{bmatrix}$$

$$\rho_{M,11} = \begin{bmatrix} 0.3090 & 0.2536 - 0.0020i & -0.0236 + 0.2387i & 0.0049 - 0.2545i \\ 0.2536 + 0.0020i & 0.2169 & -0.0266 + 0.2206i & 0.0128 - 0.2344i \\ -0.0236 - 0.2387i & -0.0266 - 0.2206i & 0.2625 & -0.2126 - 0.0164i \\ 0.0049 + 0.2545i & 0.0128 + 0.2344i & -0.2126 + 0.0164i & 0.2116 \end{bmatrix}$$

$$\rho_{M,12} = \begin{bmatrix} 0.2210 & -0.2802 - 0.0510i & -0.0475 - 0.2454i & -0.0166 - 0.2218i \\ -0.2802 + 0.0510i & 0.2512 & 0.0112 + 0.2530i & 0.0076 + 0.2359i \\ -0.0475 + 0.2454i & 0.0112 - 0.2530i & 0.1993 & 0.1977 + 0.0841i \\ -0.0166 + 0.2218i & 0.0076 - 0.2359i & 0.1977 - 0.0841i & 0.3285 \end{bmatrix}$$

$$\rho_{M,13} = \begin{bmatrix} 0.3059 & -0.2612-0.0147i & 0.0146+0.2246i & 0.0422+0.2544i \\ -0.2612+0.0147i & 0.1762 & -0.0175-0.2085i & -0.0342-0.2370i \\ 0.0146-0.2246i & -0.0175+0.2085i & 0.2792 & 0.1911+0.0008i \\ 0.0422-0.2544i & -0.0342+0.2370i & 0.1911-0.0008i & 0.2387 \end{bmatrix}$$

$$\rho_{M,14} = \begin{bmatrix} 0.4095 & -0.0753-0.3984i & -0.1351+0.1251i & 0.1159+0.0932i \\ -0.0753+0.3984i & 0.3649 & -0.0998-0.1619i & -0.0991+0.1004i \\ -0.1351-0.1251i & -0.0998+0.1619i & 0.1005 & -0.0384-0.1226i \\ 0.1159-0.0932i & -0.0991-0.1004i & -0.0384+0.1226i & 0.1251 \end{bmatrix}$$

$$\rho_{M,15} = \begin{bmatrix} 0.5590 & 0.1688+0.4115i & -0.0750+0.0461i & -0.0342-0.0741i \\ 0.1688-0.4115i & 0.3472 & 0.0472+0.0803i & -0.0795+0.0198i \\ -0.0750-0.0461i & 0.0472-0.0803i & 0.0258 & 0.0397+0.0409i \\ -0.0342+0.0741i & -0.0795-0.0198i & 0.0397-0.0409i & 0.0680 \end{bmatrix}$$

$$\rho_{M,16} = \begin{bmatrix} 0.0095 & -0.0057-0.1331i & -0.0030+0.0330i & -0.0239-0.0074i \\ -0.0057+0.1331i & 0.6278 & -0.3716-0.0931i & -0.0152-0.0669i \\ -0.0030-0.0330i & -0.3716+0.0931i & 0.3255 & 0.0919+0.0219i \\ -0.0239+0.0074i & -0.0152+0.0669i & 0.0919-0.0219i & 0.0372 \end{bmatrix}$$

$$\rho_{M,17} = \begin{bmatrix} 0.0219 & -0.0122-0.1428i & -0.0034+0.0132i & -0.0230-0.0034i \\ -0.0122+0.1428i & 0.7250 & -0.3510-0.0873i & -0.0176-0.0751i \\ -0.0034-0.0132i & -0.3510+0.0873i & 0.2151 & 0.0860+0.0050i \\ -0.0230+0.0034i & -0.0176+0.0751i & 0.0860-0.0050i & 0.0379 \end{bmatrix}$$

D. Optimal tomography

$$\rho_{O,1} = \begin{bmatrix} 0.4879 & -0.0194+0.0278i & 0.0045+0.0413i & -0.4760-0.0086i \\ -0.0194-0.0278i & 0.0054 & -0.0112-0.0003i & 0.0336+0.0064i \\ 0.0045-0.0413i & -0.0112+0.0003i & 0.0225 & -0.0033+0.0768i \\ -0.4760+0.0086i & 0.0336-0.0064i & -0.0033-0.0768i & 0.4842 \end{bmatrix}$$

$$\rho_{O,2} = \begin{bmatrix} 0.4748 & 0.0191-0.0443i & 0.0193-0.0130i & 0.4781+0.0019i \\ 0.0191+0.0443i & 0.0107 & 0.0026+0.0001i & 0.0200+0.0379i \\ 0.0193+0.0130i & 0.0026-0.0001i & 0.0079 & 0.0111-0.0464i \\ 0.4781-0.0019i & 0.0200-0.0379i & 0.0111+0.0464i & 0.5065 \end{bmatrix}$$

$$\rho_{O,3} = \begin{bmatrix} 0.5221 & 0.0816-0.0442i & 0.0847-0.0240i & 0.0530+0.4532i \\ 0.0816+0.0442i & 0.0213 & -0.0020-0.0026i & -0.0463+0.0584i \\ 0.0847+0.0240i & -0.0020+0.0026i & 0.0112 & 0.0095+0.0545i \\ 0.0530-0.4532i & -0.0463-0.0584i & 0.0095-0.0545i & 0.4454 \end{bmatrix}$$

$$\rho_{O,4} = \begin{bmatrix} 0.2807 & 0.2569+0.0406i & 0.0456-0.2118i & 0.0297+0.2155i \\ 0.2569-0.0406i & 0.2543 & 0.1047-0.1856i & -0.0304+0.2331i \\ 0.0456+0.2118i & 0.1047+0.1856i & 0.1256 & -0.2027-0.0279i \\ 0.0297-0.2155i & -0.0304-0.2331i & -0.2027+0.0279i & 0.3394 \end{bmatrix}$$

$$\rho_{O,5} = \begin{bmatrix} 0.0260 & -0.0969+0.0186i & 0.0380+0.0730i & 0.0017-0.0078i \\ -0.0969-0.0186i & 0.5035 & 0.1813-0.4222i & 0.0051-0.0374i \\ 0.0380-0.0730i & 0.1813+0.4222i & 0.4676 & -0.0051-0.0057i \\ 0.0017+0.0078i & 0.0051+0.0374i & -0.0051+0.0057i & 0.0029 \end{bmatrix}$$

$$\rho_{O,6} = \begin{bmatrix} 0.0367 & 0.0502+0.0923i & 0.0734+0.0820i & 0.0102-0.0178i \\ 0.0502-0.0923i & 0.4500 & 0.4460-0.0748i & -0.0288-0.0230i \\ 0.0734-0.0820i & 0.4460+0.0748i & 0.5064 & -0.0430-0.0023i \\ 0.0102+0.0178i & -0.0288+0.0230i & -0.0430+0.0023i & 0.0069 \end{bmatrix}$$

$$\rho_{O,7} = \begin{bmatrix} 0.0062 & 0.0557-0.0422i & 0.0047+0.0036i & -0.0072+0.0029i \\ 0.0557+0.0422i & 0.9818 & 0.0006+0.0380i & -0.0441-0.1031i \\ 0.0047-0.0036i & 0.0006-0.0380i & 0.0032 & -0.0049+0.0027i \\ -0.0072-0.0029i & -0.0441+0.1031i & -0.0049-0.0027i & 0.0087 \end{bmatrix}$$

$$\rho_{O,8} = \begin{bmatrix} 0.5550 & -0.0622+0.0204i & 0.0308-0.0306i & -0.0569-0.4699i \\ -0.0622-0.0204i & 0.0135 & 0.0053-0.0015i & -0.0050+0.0533i \\ 0.0308+0.0306i & 0.0053+0.0015i & 0.0043 & -0.0039-0.0220i \\ -0.0569+0.4699i & -0.0050-0.0533i & -0.0039+0.0220i & 0.4271 \end{bmatrix}$$

$$\rho_{O,9} = \begin{bmatrix} 0.0089 & -0.0219-0.0731i & -0.0027+0.0214i & -0.0130+0.0040i \\ -0.0219+0.0731i & 0.5684 & -0.4492+0.0011i & -0.0065-0.0551i \\ -0.0027-0.0214i & -0.4492-0.0011i & 0.4209 & -0.0068-0.0044i \\ -0.0130-0.0040i & -0.0065+0.0551i & -0.0068+0.0044i & 0.0018 \end{bmatrix}$$

$$\rho_{O,10} = \begin{bmatrix} 0.0058 & 0.0406-0.0232i & 0.0029+0.0025i & -0.0009+0.0092i \\ 0.0406+0.0232i & 0.5133 & -0.0158+0.4665i & -0.0279-0.0088i \\ 0.0029-0.0025i & -0.0158-0.4665i & 0.4753 & 0.0465+0.0408i \\ -0.0009-0.0092i & -0.0279+0.0088i & 0.0465-0.0408i & 0.0056 \end{bmatrix}$$

$$\rho_{O,11} = \begin{bmatrix} 0.2918 & 0.2395-0.0021i & -0.0242+0.2258i & 0.0050-0.2663i \\ 0.2395+0.0021i & 0.1992 & -0.0274+0.2259i & 0.0132-0.2382i \\ -0.0242-0.2258i & -0.0274-0.2259i & 0.2948 & -0.2469-0.0169i \\ 0.0050+0.2663i & 0.0132+0.2382i & -0.2469+0.0169i & 0.2142 \end{bmatrix}$$

$$\rho_{O,12} = \begin{bmatrix} 0.1923 & -0.2541-0.0502i & -0.0467-0.2470i & -0.0163-0.2401i \\ -0.2541+0.0502i & 0.3002 & 0.0111+0.2257i & 0.0075+0.2518i \\ -0.0467+0.2470i & 0.0111-0.2257i & 0.2127 & 0.2396+0.0827i \\ -0.0163+0.2401i & 0.0075-0.2518i & 0.2396-0.0827i & 0.2947 \end{bmatrix}$$

$$\rho_{O,13} = \begin{bmatrix} 0.3064 & -0.2359-0.0153i & 0.0152+0.2270i & 0.0439+0.2918i \\ -0.2359+0.0153i & 0.1884 & -0.0182-0.1992i & -0.0356-0.2367i \\ 0.0152-0.2270i & -0.0182+0.1992i & 0.2840 & 0.2487+0.0009i \\ 0.0439-0.2918i & -0.0356+0.2367i & 0.2487-0.0009i & 0.2211 \end{bmatrix}$$

$$\rho_{O,14} = \begin{bmatrix} 0.3803 & -0.0814-0.3887i & -0.1318+0.1051i & 0.1131+0.0665i \\ -0.0814+0.3887i & 0.3748 & -0.0973-0.1265i & -0.0967+0.1000i \\ -0.1318-0.1051i & -0.0973+0.1265i & 0.1345 & -0.0398-0.1196i \\ 0.1131-0.0665i & -0.0967-0.1000i & -0.0398+0.1196i & 0.1104 \end{bmatrix}$$

$$\rho_{O,15} = \begin{bmatrix} 0.5276 & 0.1680 + 0.4105i & -0.0748 + 0.0527i & -0.0341 - 0.0538i \\ 0.1680 - 0.4105i & 0.3819 & 0.0471 + 0.0874i & -0.0793 + 0.0196i \\ -0.0748 - 0.0527i & 0.0471 - 0.0874i & 0.0478 & 0.0094 + 0.0408i \\ -0.0341 + 0.0538i & -0.0793 - 0.0196i & 0.0094 - 0.0408i & 0.0427 \end{bmatrix}$$

$$\rho_{O,16} = \begin{bmatrix} 0.0207 & -0.0261 - 0.1296i & -0.0029 + 0.0401i & -0.0233 - 0.0012i \\ -0.0261 + 0.1296i & 0.6312 & -0.3619 - 0.0096i & -0.0148 - 0.0912i \\ -0.0029 - 0.0401i & -0.3619 + 0.0096i & 0.3450 & 0.0080 + 0.0213i \\ -0.0233 + 0.0012i & -0.0148 + 0.0912i & 0.0080 - 0.0213i & 0.0031 \end{bmatrix}$$

$$\rho_{O,17} = \begin{bmatrix} 0.0202 & -0.0269 - 0.1385i & -0.0033 + 0.0486i & -0.0223 + 0.0155i \\ -0.0269 + 0.1385i & 0.7415 & -0.3405 + 0.0102i & -0.0171 - 0.0985i \\ -0.0033 - 0.0486i & -0.3405 - 0.0102i & 0.2341 & 0.0106 + 0.0049i \\ -0.0223 - 0.0155i & -0.0171 + 0.0985i & 0.0106 - 0.0049i & 0.0042 \end{bmatrix}$$

E. Pauli matrices based tomography

$$\rho_{P,1} = \begin{bmatrix} 0.4879 & -0.0194 + 0.0278i & 0.0045 + 0.0413i & -0.4516 - 0.0735i \\ -0.0194 - 0.0278i & 0.0054 & -0.0053 + 0.0223i & 0.0336 + 0.0064i \\ 0.0045 - 0.0413i & -0.0053 - 0.0223i & 0.0225 & -0.0033 + 0.0768i \\ -0.4516 + 0.0735i & 0.0336 - 0.0064i & -0.0033 - 0.0768i & 0.4842 \end{bmatrix}$$

$$\rho_{P,2} = \begin{bmatrix} 0.4748 & 0.0191 - 0.0443i & 0.0193 - 0.0130i & 0.4662 + 0.0508i \\ 0.0191 + 0.0443i & 0.0107 & -0.0000 - 0.0216i & 0.0200 + 0.0379i \\ 0.0193 + 0.0130i & -0.0000 + 0.0216i & 0.0079 & 0.0111 - 0.0464i \\ 0.4662 - 0.0508i & 0.0200 - 0.0379i & 0.0111 + 0.0464i & 0.5065 \end{bmatrix}$$

$$\rho_{P,3} = \begin{bmatrix} 0.5221 & 0.0816 - 0.0442i & 0.0847 - 0.0240i & -0.0026 + 0.4523i \\ 0.0816 + 0.0442i & 0.0213 & 0.0509 - 0.0035i & -0.0463 + 0.0584i \\ 0.0847 + 0.0240i & 0.0509 + 0.0035i & 0.0112 & 0.0095 + 0.0545i \\ -0.0026 - 0.4523i & -0.0463 - 0.0584i & 0.0095 - 0.0545i & 0.4454 \end{bmatrix}$$

$$\rho_{P,4} = \begin{bmatrix} 0.2807 & 0.2569 + 0.0406i & 0.0456 - 0.2118i & -0.0623 + 0.2607i \\ 0.2569 - 0.0406i & 0.2543 & 0.0401 - 0.1770i & -0.0304 + 0.2331i \\ 0.0456 + 0.2118i & 0.0401 + 0.1770i & 0.1256 & -0.2027 - 0.0279i \\ -0.0623 - 0.2607i & -0.0304 - 0.2331i & -0.2027 + 0.0279i & 0.3394 \end{bmatrix}$$

$$\rho_{P,5} = \begin{bmatrix} 0.0260 & -0.0969 + 0.0186i & 0.0380 + 0.0730i & 0.0156 + 0.0018i \\ -0.0969 - 0.0186i & 0.5035 & 0.0389 - 0.4553i & 0.0051 - 0.0374i \\ 0.0380 - 0.0730i & 0.0389 + 0.4553i & 0.4676 & -0.0051 - 0.0057i \\ 0.0156 - 0.0018i & 0.0051 + 0.0374i & -0.0051 + 0.0057i & 0.0029 \end{bmatrix}$$

$$\rho_{P,6} = \begin{bmatrix} 0.0367 & 0.0502 + 0.0923i & 0.0734 + 0.0820i & -0.0044 + 0.0326i \\ 0.0502 - 0.0923i & 0.4500 & 0.4397 - 0.0152i & -0.0288 - 0.0230i \\ 0.0734 - 0.0820i & 0.4397 + 0.0152i & 0.5064 & -0.0430 - 0.0023i \\ -0.0044 - 0.0326i & -0.0288 + 0.0230i & -0.0430 + 0.0023i & 0.0069 \end{bmatrix}$$

$$\rho_{P,7} = \begin{bmatrix} 0.0062 & 0.0557 - 0.0422i & 0.0047 + 0.0036i & -0.0086 - 0.0005i \\ 0.0557 + 0.0422i & 0.9818 & 0.0283 + 0.0662i & -0.0441 - 0.1031i \\ 0.0047 - 0.0036i & 0.0283 - 0.0662i & 0.0032 & -0.0049 + 0.0027i \\ -0.0086 + 0.0005i & -0.0441 + 0.1031i & -0.0049 - 0.0027i & 0.0087 \end{bmatrix}$$

$$\rho_{P,8} = \begin{bmatrix} 0.5550 & -0.0622+0.0204i & 0.0308-0.0306i & 0.0026-0.4514i \\ -0.0622-0.0204i & 0.0135 & -0.0451+0.0076i & -0.0050+0.0533i \\ 0.0308+0.0306i & -0.0451-0.0076i & 0.0043 & -0.0039-0.0220i \\ 0.0026+0.4514i & -0.0050-0.0533i & -0.0039+0.0220i & 0.4271 \end{bmatrix}$$

$$\rho_{P,9} = \begin{bmatrix} 0.0089 & -0.0219-0.0731i & -0.0027+0.0214i & -0.0215-0.0387i \\ -0.0219+0.0731i & 0.5684 & -0.4398-0.0689i & -0.0065-0.0551i \\ -0.0027-0.0214i & -0.4398+0.0689i & 0.4209 & -0.0068-0.0044i \\ -0.0215+0.0387i & -0.0065+0.0551i & -0.0068+0.0044i & 0.0018 \end{bmatrix}$$

$$\rho_{P,10} = \begin{bmatrix} 0.0058 & 0.0406-0.0232i & 0.0029+0.0025i & -0.0090-0.0064i \\ 0.0406+0.0232i & 0.5133 & 0.0500+0.4689i & -0.0279-0.0088i \\ 0.0029-0.0025i & 0.0500-0.4689i & 0.4753 & 0.0465+0.0408i \\ -0.0090+0.0064i & -0.0279+0.0088i & 0.0465-0.0408i & 0.0056 \end{bmatrix}$$

$$\rho_{P,11} = \begin{bmatrix} 0.2918 & 0.2395-0.0021i & -0.0242+0.2258i & 0.0052-0.2556i \\ 0.2395+0.0021i & 0.1992 & -0.0291+0.2209i & 0.0132-0.2382i \\ -0.0242-0.2258i & -0.0291-0.2209i & 0.2948 & -0.2469-0.0169i \\ 0.0052+0.2556i & 0.0132+0.2382i & -0.2469+0.0169i & 0.2142 \end{bmatrix}$$

$$\rho_{P,12} = \begin{bmatrix} 0.1923 & -0.2541-0.0502i & -0.0467-0.2470i & 0.0350-0.2186i \\ -0.2541+0.0502i & 0.3002 & 0.0458+0.2493i & 0.0075+0.2518i \\ -0.0467+0.2470i & 0.0458-0.2493i & 0.2127 & 0.2396+0.0827i \\ 0.0350+0.2186i & 0.0075-0.2518i & 0.2396-0.0827i & 0.2947 \end{bmatrix}$$

$$\rho_{P,13} = \begin{bmatrix} 0.3064 & -0.2359-0.0153i & 0.0152+0.2270i & 0.0170+0.2694i \\ -0.2359+0.0153i & 0.1884 & 0.0105-0.2216i & -0.0356-0.2367i \\ 0.0152-0.2270i & 0.0105+0.2216i & 0.2840 & 0.2487+0.0009i \\ 0.0170-0.2694i & -0.0356+0.2367i & 0.2487-0.0009i & 0.2211 \end{bmatrix}$$

$$\rho_{P,14} = \begin{bmatrix} 0.3803 & -0.0814-0.3887i & -0.1318+0.1051i & 0.1286+0.0801i \\ -0.0814+0.3887i & 0.3748 & -0.0803-0.1471i & -0.0967+0.1000i \\ -0.1318-0.1051i & -0.0803+0.1471i & 0.1345 & -0.0398-0.1196i \\ 0.1286-0.0801i & -0.0967-0.1000i & -0.0398+0.1196i & 0.1104 \end{bmatrix}$$

$$\rho_{P,15} = \begin{bmatrix} 0.5276 & 0.1680+0.4105i & -0.0748+0.0527i & -0.0633-0.0764i \\ 0.1680-0.4105i & 0.3819 & 0.0050+0.0826i & -0.0793+0.0196i \\ -0.0748-0.0527i & 0.0050-0.0826i & 0.0478 & 0.0094+0.0408i \\ -0.0633+0.0764i & -0.0793-0.0196i & 0.0094-0.0408i & 0.0427 \end{bmatrix}$$

$$\rho_{P,16} = \begin{bmatrix} 0.0207 & -0.0261-0.1296i & -0.0029+0.0401i & -0.0181-0.0374i \\ -0.0261+0.1296i & 0.6312 & -0.4367-0.0605i & -0.0148-0.0912i \\ -0.0029-0.0401i & -0.4367+0.0605i & 0.3450 & 0.0080+0.0213i \\ -0.0181+0.0374i & -0.0148+0.0912i & 0.0080-0.0213i & 0.0031 \end{bmatrix}$$

$$\rho_{P,17} = \begin{bmatrix} 0.0202 & -0.0269-0.1385i & -0.0033+0.0486i & -0.0181-0.0334i \\ -0.0269+0.1385i & 0.7415 & -0.3803-0.0547i & -0.0171-0.0985i \\ -0.0033-0.0486i & -0.3803+0.0547i & 0.2341 & 0.0106+0.0049i \\ -0.0181+0.0334i & -0.0171+0.0985i & 0.0106-0.0049i & 0.0042 \end{bmatrix}$$

II. COEFFICIENT MATRICES

All the analyzed tomographies are based on solving the linear-system problem

$$Ax = b,$$

where A is the *coefficient matrix*, b is the *observation vector*, and $x = \mathrm{vec}(\rho)$ is a real vector describing the unknown state ρ , i.e.,

$$x = \mathrm{vec}(\rho) = [\rho_{11}, \mathrm{Re}\rho_{12}, \mathrm{Im}\rho_{12}, \mathrm{Re}\rho_{13}, \mathrm{Im}\rho_{13}, ..., \rho_{44}]^T.$$

Thus, a two-qubit density matrix ρ is represented as a real vector $x = (x_1, ..., x_{16})$ with its elements given as follows

$$\rho(x) = \begin{bmatrix} x_1 & x_2 + ix_3 & x_4 + ix_5 & x_6 + ix_7 \\ x_2 - ix_3 & x_8 & x_9 + ix_{10} & x_{11} + ix_{12} \\ x_4 - ix_5 & x_9 - ix_{10} & x_{13} & x_{14} + ix_{15} \\ x_6 - ix_7 & x_{11} - ix_{12} & x_{14} - ix_{15} & x_{16} \end{bmatrix}.$$

The coefficient matrices depend on the choice of the equations used for reconstructing a given density matrix. Below we list the transposed (for typographic reasons) coefficient matrices for the four analyzed tomographic protocols:

$$A_P^T = \begin{bmatrix} 0&0&0&0&0&2&0&0&2&0&0&0&0&0&0&0 \\ 0&0&0&0&0&0&-2&0&0&2&0&0&0&0&0&0 \\ 0&0&0&2&0&0&0&0&0&0&-2&0&0&0&0&0 \\ 0&0&0&2&0&0&0&0&0&0&2&0&0&0&0&0 \\ 0&0&0&0&0&0&-2&0&0&-2&0&0&0&0&0&0 \\ 0&0&0&0&0&-2&0&0&2&0&0&0&0&0&0&0 \\ 0&0&0&0&-2&0&0&0&0&0&0&2&0&0&0&0 \\ 0&0&0&0&-2&0&0&0&0&0&0&-2&0&0&0&0 \\ 0&2&0&0&0&0&0&0&0&0&0&0&0&-2&0&0 \\ 0&0&-2&0&0&0&0&0&0&0&0&0&0&0&2&0 \\ 1&0&0&0&0&0&0&-1&0&0&0&0&-1&0&0&1 \\ 1&0&0&0&0&0&0&1&0&0&0&0&-1&0&0&-1 \\ 0&2&0&0&0&0&0&0&0&0&0&0&0&2&0&0 \\ 0&0&-2&0&0&0&0&0&0&0&0&0&0&0&-2&0 \\ 1&0&0&0&0&0&0&-1&0&0&0&0&1&0&0&-1 \\ 1&0&0&0&0&0&0&1&0&0&0&0&1&0&0&1 \end{bmatrix}$$

$$A_S^T = \frac{1}{4}\begin{bmatrix}
4 & 0 & 0 & 0 & 0 & 0 & 0 & 0 & 0 & 0 & 0 & 0 & 0 & 0 & 0 & 0 \\
0 & 0 & 0 & 0 & 0 & 0 & 0 & 4 & 0 & 0 & 0 & 0 & 0 & 0 & 0 & 0 \\
2 & 4 & 0 & 0 & 0 & 0 & 0 & 2 & 0 & 0 & 0 & 0 & 0 & 0 & 0 & 0 \\
2 & -4 & 0 & 0 & 0 & 0 & 0 & 2 & 0 & 0 & 0 & 0 & 0 & 0 & 0 & 0 \\
2 & 0 & -4 & 0 & 0 & 0 & 0 & 2 & 0 & 0 & 0 & 0 & 0 & 0 & 0 & 0 \\
2 & 0 & 4 & 0 & 0 & 0 & 0 & 2 & 0 & 0 & 0 & 0 & 0 & 0 & 0 & 0 \\
0 & 0 & 0 & 0 & 0 & 0 & 0 & 0 & 0 & 0 & 0 & 0 & 4 & 0 & 0 & 0 \\
0 & 0 & 0 & 0 & 0 & 0 & 0 & 0 & 0 & 0 & 0 & 0 & 0 & 0 & 0 & 4 \\
0 & 0 & 0 & 0 & 0 & 0 & 0 & 0 & 0 & 0 & 0 & 0 & 2 & 4 & 0 & 2 \\
0 & 0 & 0 & 0 & 0 & 0 & 0 & 0 & 0 & 0 & 0 & 0 & 2 & -4 & 0 & 2 \\
0 & 0 & 0 & 0 & 0 & 0 & 0 & 0 & 0 & 0 & 0 & 0 & 2 & 0 & -4 & 2 \\
0 & 0 & 0 & 0 & 0 & 0 & 0 & 0 & 0 & 0 & 0 & 0 & 2 & 0 & 4 & 2 \\
2 & 0 & 0 & 4 & 0 & 0 & 0 & 0 & 0 & 0 & 0 & 0 & 2 & 0 & 0 & 0 \\
0 & 0 & 0 & 0 & 0 & 0 & 0 & 2 & 0 & 0 & 4 & 0 & 0 & 0 & 0 & 2 \\
1 & 2 & 0 & 2 & 0 & 2 & 0 & 1 & 2 & 0 & 2 & 0 & 1 & 2 & 0 & 1 \\
1 & -2 & 0 & 2 & 0 & -2 & 0 & 1 & -2 & 0 & 2 & 0 & 1 & -2 & 0 & 1 \\
1 & 0 & -2 & 2 & 0 & 0 & -2 & 1 & 0 & 2 & 2 & 0 & 1 & 0 & -2 & 1 \\
1 & 0 & 2 & 2 & 0 & 0 & 2 & 1 & 0 & -2 & 2 & 0 & 1 & 0 & 2 & 1 \\
2 & 0 & 0 & -4 & 0 & 0 & 0 & 0 & 0 & 0 & 0 & 0 & 2 & 0 & 0 & 0 \\
0 & 0 & 0 & 0 & 0 & 0 & 0 & 2 & 0 & 0 & -4 & 0 & 0 & 0 & 0 & 2 \\
1 & 2 & 0 & -2 & 0 & -2 & 0 & 1 & -2 & 0 & -2 & 0 & 1 & 2 & 0 & 1 \\
1 & -2 & 0 & -2 & 0 & 2 & 0 & 1 & 2 & 0 & -2 & 0 & 1 & -2 & 0 & 1 \\
1 & 0 & -2 & -2 & 0 & 0 & 2 & 1 & 0 & -2 & -2 & 0 & 1 & 0 & -2 & 1 \\
1 & 0 & 2 & -2 & 0 & 0 & -2 & 1 & 0 & 2 & -2 & 0 & 1 & 0 & 2 & 1 \\
2 & 0 & 0 & 0 & -4 & 0 & 0 & 0 & 0 & 0 & 0 & 0 & 2 & 0 & 0 & 0 \\
0 & 0 & 0 & 0 & 0 & 0 & 0 & 2 & 0 & 0 & 0 & -4 & 0 & 0 & 0 & 2 \\
1 & 2 & 0 & 0 & -2 & 0 & -2 & 1 & 0 & -2 & 0 & -2 & 1 & 2 & 0 & 1 \\
1 & -2 & 0 & 0 & -2 & 0 & 2 & 1 & 0 & 2 & 0 & -2 & 1 & -2 & 0 & 1 \\
1 & 0 & -2 & 0 & -2 & -2 & 0 & 1 & 2 & 0 & 0 & -2 & 1 & 0 & -2 & 1 \\
1 & 0 & 2 & 0 & -2 & 2 & 0 & 1 & -2 & 0 & 0 & -2 & 1 & 0 & 2 & 1 \\
2 & 0 & 0 & 0 & 4 & 0 & 0 & 0 & 0 & 0 & 0 & 0 & 2 & 0 & 0 & 0 \\
0 & 0 & 0 & 0 & 0 & 0 & 0 & 2 & 0 & 0 & 0 & 4 & 0 & 0 & 0 & 2 \\
1 & 2 & 0 & 0 & 2 & 0 & 2 & 1 & 0 & 2 & 0 & 2 & 1 & 2 & 0 & 1 \\
1 & -2 & 0 & 0 & 2 & 0 & -2 & 1 & 0 & -2 & 0 & 2 & 1 & -2 & 0 & 1 \\
1 & 0 & -2 & 0 & 2 & 2 & 0 & 1 & -2 & 0 & 0 & 2 & 1 & 0 & -2 & 1 \\
1 & 0 & 2 & 0 & 2 & -2 & 0 & 1 & 2 & 0 & 0 & 2 & 1 & 0 & 2 & 1
\end{bmatrix}$$

$$A_J^T = \frac{1}{4}\begin{bmatrix}
4 & 0 & 0 & 0 & 0 & 0 & 0 & 0 & 0 & 0 & 0 & 0 & 0 & 0 & 0 & 0 \\
0 & 0 & 0 & 0 & 0 & 0 & 0 & 4 & 0 & 0 & 0 & 0 & 0 & 0 & 0 & 0 \\
2 & 4 & 0 & 0 & 0 & 0 & 0 & 2 & 0 & 0 & 0 & 0 & 0 & 0 & 0 & 0 \\
2 & 0 & -4 & 0 & 0 & 0 & 0 & 2 & 0 & 0 & 0 & 0 & 0 & 0 & 0 & 0 \\
0 & 0 & 0 & 0 & 0 & 0 & 0 & 0 & 0 & 0 & 0 & 0 & 4 & 0 & 0 & 0 \\
0 & 0 & 0 & 0 & 0 & 0 & 0 & 0 & 0 & 0 & 0 & 0 & 0 & 0 & 0 & 4 \\
0 & 0 & 0 & 0 & 0 & 0 & 0 & 0 & 0 & 0 & 0 & 0 & 2 & 4 & 0 & 2 \\
0 & 0 & 0 & 0 & 0 & 0 & 0 & 0 & 0 & 0 & 0 & 0 & 2 & 0 & -4 & 2 \\
2 & 0 & 0 & 0 & 4 & 0 & 0 & 0 & 0 & 0 & 0 & 0 & 2 & 0 & 0 & 0 \\
0 & 0 & 0 & 0 & 0 & 0 & 0 & 2 & 0 & 0 & 0 & 4 & 0 & 0 & 0 & 2 \\
1 & 2 & 0 & 0 & 2 & 0 & 2 & 1 & 0 & 2 & 0 & 2 & 1 & 2 & 0 & 1 \\
1 & 0 & -2 & 0 & 2 & 2 & 0 & 1 & -2 & 0 & 0 & 2 & 1 & 0 & -2 & 1 \\
2 & 0 & 0 & 4 & 0 & 0 & 0 & 0 & 0 & 0 & 0 & 0 & 2 & 0 & 0 & 0 \\
0 & 0 & 0 & 0 & 0 & 0 & 0 & 2 & 0 & 0 & 4 & 0 & 0 & 0 & 0 & 2 \\
1 & 2 & 0 & 2 & 0 & 2 & 0 & 1 & 2 & 0 & 2 & 0 & 1 & 2 & 0 & 1 \\
1 & 0 & 2 & 2 & 0 & 0 & 2 & 1 & 0 & -2 & 2 & 0 & 1 & 0 & 2 & 1
\end{bmatrix}$$

$$
A_M^T = \frac{1}{4}\begin{bmatrix}
2 & 0 & 0 & 4 & 0 & 0 & 0 & 0 & 0 & 0 & 0 & 0 & 2 & 0 & 0 & 0 \\
0 & 0 & 0 & 0 & 0 & 0 & 0 & 2 & 0 & 0 & 4 & 0 & 0 & 0 & 0 & 2 \\
2 & 0 & 0 & -4 & 0 & 0 & 0 & 0 & 0 & 0 & 0 & 0 & 2 & 0 & 0 & 0 \\
0 & 0 & 0 & 0 & 0 & 0 & 0 & 2 & 0 & 0 & -4 & 0 & 0 & 0 & 0 & 2 \\
1 & 2 & 0 & 0 & -2 & 0 & -2 & 1 & 0 & -2 & 0 & -2 & 1 & 2 & 0 & 1 \\
1 & -2 & 0 & 0 & -2 & 0 & 2 & 1 & 0 & 2 & 0 & -2 & 1 & -2 & 0 & 1 \\
1 & 2 & 0 & 0 & 2 & 0 & 2 & 1 & 0 & 2 & 0 & 2 & 1 & 2 & 0 & 1 \\
1 & -2 & 0 & 0 & 2 & 0 & -2 & 1 & 0 & -2 & 0 & 2 & 1 & -2 & 0 & 1 \\
0 & 0 & 0 & 0 & 0 & 0 & 0 & 0 & 0 & 0 & 0 & 0 & 2 & 0 & 4 & 2 \\
0 & 0 & 0 & 0 & 0 & 0 & 0 & 0 & 0 & 0 & 0 & 0 & 2 & 0 & -4 & 2 \\
2 & 0 & 4 & 0 & 0 & 0 & 0 & 2 & 0 & 0 & 0 & 0 & 0 & 0 & 0 & 0 \\
2 & 0 & -4 & 0 & 0 & 0 & 0 & 2 & 0 & 0 & 0 & 0 & 0 & 0 & 0 & 0 \\
2 & 0 & 0 & 0 & 0 & 4 & 0 & 0 & 0 & 0 & 0 & 0 & 0 & 0 & 0 & 2 \\
2 & 0 & 0 & 0 & 0 & -4 & 0 & 0 & 0 & 0 & 0 & 0 & 0 & 0 & 0 & 2 \\
0 & 0 & 0 & 0 & 0 & 0 & 0 & 2 & 4 & 0 & 0 & 0 & 2 & 0 & 0 & 0 \\
0 & 0 & 0 & 0 & 0 & 0 & 0 & 2 & -4 & 0 & 0 & 0 & 2 & 0 & 0 & 0 \\
1 & 2 & 0 & 0 & 2 & 0 & -2 & 1 & 0 & 2 & 0 & -2 & 1 & -2 & 0 & 1 \\
1 & -2 & 0 & 0 & -2 & 0 & -2 & 1 & 0 & 2 & 0 & 2 & 1 & 2 & 0 & 1 \\
1 & -2 & 0 & 0 & 2 & 0 & 2 & 1 & 0 & -2 & 0 & -2 & 1 & 2 & 0 & 1 \\
1 & 2 & 0 & 0 & -2 & 0 & 2 & 1 & 0 & -2 & 0 & 2 & 1 & -2 & 0 & 1
\end{bmatrix}
$$

$$
A_O^T = \begin{bmatrix}
1 & 0 & 0 & 0 & 0 & 0 & 0 & 0 & 0 & 0 & 0 & 0 & 0 & 0 & 0 & 0 \\
0 & 0 & 0 & 0 & 0 & 0 & 0 & 1 & 0 & 0 & 0 & 0 & 0 & 0 & 0 & 0 \\
0 & 0 & 0 & 0 & 0 & 0 & 0 & 0 & 0 & 0 & 0 & 0 & 1 & 0 & 0 & 0 \\
0 & 0 & 0 & 0 & 0 & 0 & 0 & 0 & 0 & 0 & 0 & 0 & 0 & 0 & 0 & 1 \\
0 & 1 & 0 & 0 & 0 & 0 & 0 & 0 & 0 & 0 & 0 & 0 & 0 & 0 & 0 & 0 \\
0 & 0 & -1 & 0 & 0 & 0 & 0 & 0 & 0 & 0 & 0 & 0 & 0 & 0 & 0 & 0 \\
0 & 0 & 0 & 1 & 0 & 0 & 0 & 0 & 0 & 0 & 0 & 0 & 0 & 0 & 0 & 0 \\
0 & 0 & 0 & 0 & -1 & 0 & 0 & 0 & 0 & 0 & 0 & 0 & 0 & 0 & 0 & 0 \\
0 & 0 & 0 & 0 & 0 & 0 & 0 & 0 & 0 & 0 & 0 & 0 & 0 & 1 & 0 & 0 \\
0 & 0 & 0 & 0 & 0 & 0 & 0 & 0 & 0 & 0 & 0 & 0 & 0 & 0 & -1 & 0 \\
0 & 0 & 0 & 0 & 0 & 0 & 0 & 0 & 0 & 0 & 1 & 0 & 0 & 0 & 0 & 0 \\
0 & 0 & 0 & 0 & 0 & 0 & 0 & 0 & 0 & 0 & 0 & -1 & 0 & 0 & 0 & 0 \\
0 & 0 & 0 & 0 & 0 & 0 & 0 & 0 & 1 & 0 & 0 & 0 & 0 & 0 & 0 & 0 \\
0 & 0 & 0 & 0 & 0 & 0 & 0 & 0 & 0 & -1 & 0 & 0 & 0 & 0 & 0 & 0 \\
0 & 0 & 0 & 0 & 0 & 1 & 0 & 0 & 0 & 0 & 0 & 0 & 0 & 0 & 0 & 0 \\
0 & 0 & 0 & 0 & 0 & 0 & -1 & 0 & 0 & 0 & 0 & 0 & 0 & 0 & 0 & 0
\end{bmatrix}
$$

III. OBSERVATION VECTORS

The observation vectors correspond to photon coincidence counts. In reality we measure disturbed quantities $\bar{b} \equiv b + \delta\, b$ instead of b. The observation vectors are column vectors. For convenience we arrange them in arrays, where each column corresponds to one of the 17 reconstructed states.

$$
\bar{b} = \begin{array}{c} \\ b_1 \\ b_2 \\ \vdots \\ b_N \end{array}
\begin{array}{c} \begin{array}{cccc} \rho_1 & \rho_2 & \cdots & \rho_{17} \end{array} \\
\begin{bmatrix}
\bar{b}_{1,1} & \bar{b}_{1,2} & \cdots & \bar{b}_{1,17} \\
\bar{b}_{2,1} & \bar{b}_{2,2} & \cdots & \bar{b}_{2,17} \\
\vdots & \vdots & \ddots & \vdots \\
\bar{b}_{N,1} & \bar{b}_{N,2} & \cdots & \bar{b}_{N,17}
\end{bmatrix} \end{array}
$$

Note that the values of b listed below are not normalized and cannot be interpreted as probabilities. The elements of each vector b were registered over 5 seconds. This means that if an element of b is a sum or a difference of n projectors the measurement for each of the n projectors took $5/n$ seconds. In this way the measurements for observation vectors of the same length take the same amount of time. To obtain the frequencies we can divide these values by the total

number of photon coincidences counted or by a sum of coincidences counted for a set of projectors forming a basis. The set of such projectors is not unique. In our calculations we use the unnormalized coincidences and normalize the reconstructed density matrices.

A. Standard 36 state tomography

$$\bar{b}_S = \begin{bmatrix}
2727 & 2575 & 2844 & 1448 & 127 & 193 & 25 & 2955 & 40 & 27 & 1264 & 809 & 1231 & 2762 & 3831 & 113 & 112 \\
30 & 58 & 116 & 1312 & 2457 & 2364 & 3928 & 72 & 2555 & 2375 & 863 & 1263 & 757 & 2722 & 2773 & 3452 & 4102 \\
1244 & 1401 & 1890 & 2688 & 826 & 1483 & 2159 & 1096 & 1152 & 1386 & 2126 & 40 & 50 & 2251 & 4529 & 1758 & 2012 \\
1461 & 1194 & 1001 & 37 & 1772 & 956 & 1713 & 1758 & 1349 & 1010 & 51 & 2178 & 1945 & 3434 & 2090 & 2043 & 2310 \\
1270 & 1697 & 1865 & 1224 & 1120 & 722 & 2102 & 1407 & 1433 & 1210 & 1150 & 1200 & 1057 & 5667 & 266 & 2409 & 2782 \\
1581 & 1216 & 1383 & 1643 & 1302 & 1692 & 1764 & 1624 & 776 & 995 & 1132 & 778 & 934 & 21 & 6227 & 991 & 1250 \\
126 & 43 & 61 & 648 & 2282 & 2660 & 13 & 23 & 1892 & 2199 & 1277 & 895 & 1141 & 977 & 347 & 1887 & 1295 \\
2706 & 2747 & 2426 & 1751 & 14 & 36 & 35 & 2274 & 8 & 26 & 928 & 1240 & 888 & 802 & 310 & 17 & 23 \\
1403 & 1448 & 1248 & 113 & 1051 & 1095 & 10 & 1134 & 951 & 1399 & 35 & 2079 & 2034 & 646 & 352 & 974 & 719 \\
1440 & 1328 & 1144 & 2204 & 1101 & 1547 & 49 & 1175 & 1012 & 969 & 2174 & 63 & 36 & 1224 & 216 & 887 & 602 \\
940 & 1611 & 907 & 1238 & 1253 & 1422 & 11 & 1235 & 1018 & 1069 & 1099 & 756 & 1067 & 1769 & 9 & 852 & 664 \\
1798 & 1108 & 1501 & 950 & 1197 & 1398 & 33 & 1001 & 978 & 1447 & 953 & 1452 & 1074 & 31 & 601 & 1085 & 718 \\
1316 & 1304 & 1837 & 1250 & 1385 & 1781 & 42 & 1458 & 952 & 1172 & 1147 & 710 & 1251 & 901 & 1634 & 939 & 651 \\
1607 & 1537 & 981 & 1279 & 1149 & 920 & 1594 & 1156 & 1207 & 1065 & 991 & 1268 & 691 & 1085 & 987 & 1753 & 1986 \\
42 & 2845 & 1706 & 1231 & 1225 & 2556 & 962 & 1090 & 32 & 1482 & 996 & 1111 & 1012 & 790 & 1732 & 81 & 208 \\
2740 & 40 & 967 & 1276 & 1273 & 87 & 684 & 1568 & 2169 & 886 & 1160 & 819 & 880 & 1359 & 755 & 2663 & 2424 \\
1354 & 1497 & 107 & 25 & 25 & 1165 & 1024 & 2671 & 1050 & 2240 & 2215 & 1949 & 64 & 2083 & 131 & 1545 & 1485 \\
1298 & 1459 & 2689 & 2352 & 2356 & 1818 & 564 & 43 & 876 & 156 & 47 & 72 & 1866 & 5 & 2458 & 1053 & 938 \\
1266 & 1095 & 914 & 780 & 1014 & 1010 & 4 & 1130 & 976 & 1145 & 1357 & 1103 & 1129 & 2815 & 2720 & 971 & 688 \\
1231 & 1320 & 1485 & 1593 & 1099 & 1223 & 1947 & 1209 & 1265 & 1323 & 877 & 1205 & 977 & 2489 & 2139 & 1915 & 2175 \\
2459 & 57 & 1288 & 1250 & 791 & 28 & 1043 & 1137 & 2090 & 1251 & 1081 & 944 & 1118 & 2145 & 3266 & 2585 & 2534 \\
50 & 2308 & 1075 & 1066 & 1371 & 2133 & 923 & 1163 & 80 & 1034 & 1038 & 1332 & 1207 & 3415 & 1442 & 192 & 342 \\
818 & 1591 & 2448 & 2275 & 2158 & 947 & 875 & 22 & 1305 & 22 & 39 & 31 & 2170 & 5417 & 141 & 1841 & 1860 \\
1833 & 767 & 65 & 86 & 28 & 1098 & 949 & 2281 & 859 & 2336 & 1999 & 2091 & 28 & 38 & 4777 & 1096 & 1077 \\
1229 & 1498 & 1585 & 2277 & 712 & 772 & 2 & 1617 & 689 & 979 & 80 & 2142 & 61 & 1173 & 1991 & 601 & 314 \\
1259 & 1048 & 878 & 158 & 1535 & 1390 & 2521 & 804 & 1576 & 1348 & 2087 & 66 & 1988 & 1094 & 1532 & 2478 & 2874 \\
1743 & 924 & 123 & 1133 & 2117 & 918 & 1288 & 2314 & 1521 & 60 & 1199 & 856 & 799 & 994 & 2446 & 1994 & 2063 \\
850 & 1559 & 2345 & 1188 & 213 & 1343 & 1326 & 81 & 703 & 2252 & 866 & 1327 & 1284 & 1313 & 981 & 999 & 1180 \\
2423 & 84 & 1608 & 1713 & 1499 & 2127 & 1416 & 936 & 189 & 970 & 718 & 914 & 1206 & 2181 & 142 & 455 & 725 \\
199 & 2354 & 828 & 733 & 902 & 34 & 1028 & 1672 & 1919 & 1103 & 1253 & 1332 & 677 & 12 & 3277 & 2470 & 2414 \\
1691 & 1357 & 1323 & 92 & 1424 & 1634 & 31 & 1291 & 881 & 1002 & 2036 & 64 & 1885 & 2699 & 2757 & 1040 & 852 \\
1331 & 1459 & 1514 & 2563 & 1170 & 1148 & 1696 & 1372 & 1081 & 1267 & 23 & 2185 & 86 & 2546 & 1817 & 1481 & 1784 \\
1648 & 1243 & 2746 & 1707 & 30 & 1217 & 1114 & 42 & 991 & 2140 & 1068 & 946 & 939 & 2106 & 2968 & 1278 & 1244 \\
1327 & 1561 & 79 & 899 & 2552 & 1460 & 626 & 2535 & 1140 & 52 & 1036 & 1158 & 1040 & 3398 & 1413 & 1354 & 1335 \\
48 & 2814 & 1524 & 1198 & 1435 & 84 & 889 & 1204 & 2062 & 804 & 913 & 746 & 1349 & 5230 & 157 & 2610 & 2403 \\
2812 & 27 & 1327 & 1275 & 1066 & 2657 & 796 & 1432 & 32 & 1483 & 1151 & 1255 & 768 & 27 & 4284 & 47 & 85
\end{bmatrix}$$

The rows of the observation vector $\bar{b}_S$ correspond to the following consecutive projectors: $|HH\rangle\langle HH|$, $|HV\rangle\langle HV|$, $|HD\rangle\langle HD|$, $|HA\rangle\langle HA|$, $|HL\rangle\langle HL|$, $|HR\rangle\langle HR|$, $|VH\rangle\langle VH|$, $|VV\rangle\langle VV|$, $|VD\rangle\langle VD|$, $|VA\rangle\langle VA|$, $|VL\rangle\langle VL|$, $|VR\rangle\langle VR|$, $|DH\rangle\langle DH|$, $|DV\rangle\langle DV|$, $|DD\rangle\langle DD|$, $|DA\rangle\langle DA|$, $|DL\rangle\langle DL|$, $|DR\rangle\langle DR|$, $|AH\rangle\langle AH|$, $|AV\rangle\langle AV|$, $|AD\rangle\langle AD|$, $|AA\rangle\langle AA|$, $|AL\rangle\langle AL|$, $|AR\rangle\langle AR|$, $|LH\rangle\langle LH|$, $|LV\rangle\langle LV|$, $|LD\rangle\langle LD|$, $|LA\rangle\langle LA|$, $|LL\rangle\langle LL|$, $|LR\rangle\langle LR|$, $|RH\rangle\langle RH|$, $|RV\rangle\langle RV|$, $|RD\rangle\langle RD|$, $|RA\rangle\langle RA|$, $|RL\rangle\langle RL|$, $|RR\rangle\langle RR|$.

B. JKMW tomography

$$
\bar{b}_J = \begin{bmatrix}
2727 & 2575 & 2844 & 1448 & 127 & 193 & 25 & 2955 & 40 & 27 & 1264 & 809 & 1231 & 2762 & 3831 & 113 & 112 \\
30 & 58 & 116 & 1312 & 2457 & 2364 & 3928 & 72 & 2555 & 2375 & 863 & 1263 & 757 & 2722 & 2773 & 3452 & 4102 \\
1244 & 1401 & 1890 & 2688 & 826 & 1483 & 2159 & 1096 & 1152 & 1386 & 2126 & 40 & 50 & 2251 & 4529 & 1758 & 2012 \\
1270 & 1697 & 1865 & 1224 & 1120 & 722 & 2102 & 1407 & 1433 & 1210 & 1150 & 1200 & 1057 & 5667 & 266 & 2409 & 2782 \\
126 & 43 & 61 & 648 & 2282 & 2660 & 13 & 23 & 1892 & 2199 & 1277 & 895 & 1141 & 977 & 347 & 1887 & 1295 \\
2706 & 2747 & 2426 & 1751 & 14 & 36 & 35 & 2274 & 8 & 26 & 928 & 1240 & 888 & 802 & 310 & 17 & 23 \\
1403 & 1448 & 1248 & 113 & 1051 & 1095 & 10 & 1134 & 951 & 1399 & 35 & 2079 & 2034 & 646 & 352 & 974 & 719 \\
940 & 1611 & 907 & 1238 & 1253 & 1422 & 11 & 1235 & 1018 & 1069 & 1099 & 756 & 1067 & 1769 & 9 & 852 & 664 \\
1691 & 1357 & 1323 & 92 & 1424 & 1634 & 31 & 1291 & 881 & 1002 & 2036 & 64 & 1885 & 2699 & 2757 & 1040 & 852 \\
1331 & 1459 & 1514 & 2563 & 1170 & 1148 & 1696 & 1372 & 1081 & 1267 & 23 & 2185 & 86 & 2546 & 1817 & 1481 & 1784 \\
1648 & 1243 & 2746 & 1707 & 30 & 1217 & 1114 & 42 & 991 & 2140 & 1068 & 946 & 939 & 2106 & 2968 & 1278 & 1244 \\
48 & 2814 & 1524 & 1198 & 1435 & 84 & 889 & 1204 & 2062 & 804 & 913 & 746 & 1349 & 5230 & 157 & 2610 & 2403 \\
1316 & 1304 & 1837 & 1250 & 1385 & 1781 & 42 & 1458 & 952 & 1172 & 1147 & 710 & 1251 & 901 & 1634 & 939 & 651 \\
1607 & 1537 & 981 & 1279 & 1149 & 920 & 1594 & 1156 & 1207 & 1065 & 991 & 1268 & 691 & 1085 & 987 & 1753 & 1986 \\
42 & 2845 & 1706 & 1231 & 1225 & 2556 & 962 & 1090 & 32 & 1482 & 996 & 1111 & 1012 & 790 & 1732 & 81 & 208 \\
1298 & 1459 & 2689 & 2352 & 2356 & 1818 & 564 & 43 & 876 & 156 & 47 & 72 & 1866 & 5 & 2458 & 1053 & 938
\end{bmatrix}
$$

The rows of the observation vector $\bar{b}_J$ correspond to the following consecutive projectors: $|HH\rangle\langle HH|$, $|HV\rangle\langle HV|$, $|HD\rangle\langle HD|$, $|HL\rangle\langle HL|$, $|VH\rangle\langle VH|$, $|VV\rangle\langle VV|$, $|VD\rangle\langle VD|$, $|VL\rangle\langle VL|$, $|RH\rangle\langle RH|$, $|RV\rangle\langle RV|$, $|RD\rangle\langle RD|$, $|RL\rangle\langle RL|$, $|DH\rangle\langle DH|$, $|DV\rangle\langle DV|$, $|DD\rangle\langle DD|$, $|DR\rangle\langle DR|$.

C. MUB-based tomography

$$
\bar{b}_M = \begin{bmatrix}
1316 & 1304 & 1837 & 1250 & 1385 & 1781 & 42 & 1458 & 952 & 1172 & 1147 & 710 & 1251 & 901 & 1634 & 939 & 651 \\
1607 & 1537 & 981 & 1279 & 1149 & 920 & 1594 & 1156 & 1207 & 1065 & 991 & 1268 & 691 & 1085 & 987 & 1753 & 1986 \\
1266 & 1095 & 914 & 780 & 1014 & 1010 & 4 & 1130 & 976 & 1145 & 1357 & 1103 & 1129 & 2815 & 2720 & 971 & 688 \\
1231 & 1320 & 1485 & 1593 & 1099 & 1223 & 1947 & 1209 & 1265 & 1323 & 877 & 1205 & 977 & 2489 & 2139 & 1915 & 2175 \\
1743 & 924 & 123 & 1133 & 2117 & 918 & 1288 & 2314 & 1521 & 60 & 1199 & 856 & 799 & 994 & 2446 & 1994 & 2063 \\
850 & 1559 & 2345 & 1188 & 213 & 1343 & 1326 & 81 & 703 & 2252 & 866 & 1327 & 1284 & 1313 & 981 & 999 & 1180 \\
1648 & 1243 & 2746 & 1707 & 30 & 1217 & 1114 & 42 & 991 & 2140 & 1068 & 946 & 939 & 2106 & 2968 & 1278 & 1244 \\
1327 & 1561 & 79 & 899 & 2552 & 1460 & 626 & 2535 & 1140 & 52 & 1036 & 1158 & 1040 & 3398 & 1413 & 1354 & 1335 \\
1798 & 1108 & 1501 & 950 & 1197 & 1398 & 33 & 1001 & 978 & 1447 & 953 & 1452 & 1074 & 31 & 601 & 1085 & 718 \\
940 & 1611 & 907 & 1238 & 1253 & 1422 & 11 & 1235 & 1018 & 1069 & 1099 & 756 & 1067 & 1769 & 9 & 852 & 664 \\
1581 & 1216 & 1383 & 1643 & 1302 & 1692 & 1764 & 1624 & 776 & 995 & 1132 & 778 & 934 & 21 & 6227 & 991 & 1250 \\
1270 & 1697 & 1865 & 1224 & 1120 & 722 & 2102 & 1407 & 1433 & 1210 & 1150 & 1200 & 1057 & 5667 & 266 & 2409 & 2782 \\
97 & 5284 & 2720 & 1181 & 135 & 134 & 75 & 2337 & 16 & 23 & 1323 & 958 & 1438 & 2600 & 1867 & 23 & 21 \\
5418 & 98 & 2143 & 874 & 119 & 27 & 133 & 2943 & 133 & 31 & 1279 & 1095 & 1085 & 957 & 2362 & 277 & 268 \\
32 & 55 & 87 & 1558 & 3494 & 4939 & 1892 & 88 & 175 & 2121 & 1091 & 868 & 1002 & 826 & 1537 & 586 & 623 \\
157 & 27 & 109 & 478 & 1725 & 253 & 1887 & 32 & 4213 & 2268 & 1328 & 775 & 1148 & 2240 & 853 & 4544 & 4390 \\
2022 & 1093 & 184 & 120 & 182 & 1636 & 1011 & 2320 & 786 & 1912 & 4230 & 57 & 48 & 607 & 2921 & 944 & 945 \\
1245 & 851 & 111 & 130 & 41 & 646 & 637 & 2232 & 936 & 2583 & 52 & 4028 & 9 & 693 & 1795 & 931 & 998 \\
1404 & 1117 & 1788 & 40 & 2693 & 1246 & 990 & 60 & 1415 & 71 & 43 & 70 & 3875 & 2676 & 868 & 1920 & 1922 \\
1355 & 1652 & 3361 & 4078 & 1349 & 1156 & 613 & 115 & 862 & 81 & 13 & 84 & 54 & 2240 & 1613 & 868 & 921
\end{bmatrix}
$$

The rows of the observation vector $\bar{b}_M$ correspond to the following consecutive projectors: $|DH\rangle\langle DH|$, $|DV\rangle\langle DV|$, $|AH\rangle\langle AH|$, $|AV\rangle\langle AV|$, $|LD\rangle\langle LD|$, $|LA\rangle\langle LA|$, $|RD\rangle\langle RD|$, $|RA\rangle\langle RA|$, $|VR\rangle\langle VR|$, $|VL\rangle\langle VL|$, $|HR\rangle\langle HR|$, $|HL\rangle\langle HL|$, $|\Phi^+\rangle\langle\Phi^+|$, $|\Phi^-\rangle\langle\Phi^-|$, $|\Psi^+\rangle\langle\Psi^+|$, $|\Psi^-\rangle\langle\Psi^-|$, $\frac{1}{2}(|DL\rangle+i|AR\rangle)(\langle DL|-i\langle AR|)$, $\frac{1}{2}(|DL\rangle-i|AR\rangle)(\langle DL|+i\langle AR|)$, $\frac{1}{2}(|DR\rangle+i|AL\rangle)(\langle DR|-i\langle AL|)$, $\frac{1}{2}(|DR\rangle-i|AL\rangle)(\langle DR|+i\langle AL|)$, where $|\Phi^{\pm}\rangle = (|HH\rangle \pm |VV\rangle)/\sqrt{2}$ and $|\Psi^{\pm}\rangle = (|HV\rangle \pm |VH\rangle)/\sqrt{2}$.

D. Optimal tomography

$$
\bar{b}_O = \begin{bmatrix}
2727 & 2575 & 2844 & 1448 & 127 & 193 & 25 & 2955 & 40 & 27 & 1264 & 809 & 1231 & 2762 & 3831 & 113 & 112 \\
30 & 58 & 116 & 1312 & 2457 & 2364 & 3928 & 72 & 2555 & 2375 & 863 & 1263 & 757 & 2722 & 2773 & 3452 & 4102 \\
126 & 43 & 61 & 648 & 2282 & 2660 & 13 & 23 & 1892 & 2199 & 1277 & 895 & 1141 & 977 & 347 & 1887 & 1295 \\
2706 & 2747 & 2426 & 1751 & 14 & 36 & 35 & 2274 & 8 & 26 & 928 & 1240 & 888 & 802 & 310 & 17 & 23 \\
\overline{108} & 103 & 444 & 1325 & \overline{473} & 263 & 223 & \overline{331} & \overline{98} & 188 & 1037 & \overline{1069} & \overline{947} & \overline{591} & 1219 & \overline{142} & \overline{149} \\
\overline{155} & 240 & 241 & \overline{209} & \overline{91} & \overline{485} & 169 & \overline{108} & 328 & 107 & 9 & 211 & 61 & 2823 & \overline{2980} & 709 & 766 \\
25 & 104 & 461 & 235 & 185 & 385 & 19 & 164 & \overline{12} & 13 & \overline{105} & \overline{196} & 61 & \overline{957} & \overline{543} & \overline{16} & \overline{18} \\
\overline{231} & 70 & 131 & 1092 & \overline{356} & \overline{431} & \overline{14} & 163 & \overline{96} & \overline{11} & \overline{978} & 1039 & \overline{912} & \overline{763} & \overline{383} & \overline{219} & \overline{269} \\
\overline{18} & 60 & 52 & \overline{1045} & \overline{25} & \overline{226} & \overline{19} & \overline{20} & \overline{30} & 215 & \overline{1069} & 1008 & 999 & \overline{289} & 68 & 43 & 58 \\
\overline{429} & 251 & \overline{297} & 144 & 28 & 12 & \overline{11} & 117 & 20 & \overline{189} & 73 & \overline{348} & \overline{3} & 869 & \overline{296} & \overline{116} & \overline{27} \\
188 & 108 & \overline{252} & \overline{157} & 25 & \overline{151} & \overline{176} & \overline{26} & \overline{29} & \overline{129} & 57 & 31 & \overline{143} & \overline{702} & \overline{576} & \overline{81} & \overline{94} \\
\overline{36} & \overline{205} & \overline{318} & \overline{1202} & 182 & 121 & 412 & \overline{284} & 247 & 40 & 1032 & \overline{1059} & 951 & \overline{726} & \overline{142} & 498 & 545 \\
\overline{62} & 13 & \overline{11} & 540 & 884 & 2342 & 2 & 27 & \overline{2019} & \overline{73} & \overline{118} & 46 & \overline{73} & 706 & 341 & \overline{1979} & \overline{1883} \\
1 & \overline{0} & 14 & 957 & 2060 & 393 & \overline{151} & 7 & \overline{4} & \overline{2158} & \overline{978} & \overline{949} & 799 & 918 & \overline{634} & 52 & \overline{56} \\
\overline{2660} & 2593 & 288 & 153 & 8 & 53 & \overline{28} & \overline{302} & \overline{58} & \overline{4} & 21 & \overline{68} & 176 & 821 & \overline{247} & \overline{127} & \overline{123} \\
48 & \overline{10} & \overline{2468} & \overline{1111} & 38 & 93 & \overline{11} & 2501 & \overline{18} & \overline{42} & 1153 & 1010 & \overline{1172} & \overline{482} & 390 & 6 & \overline{85}
\end{bmatrix}
$$

The negative elements of $\bar{b}_O$ are marked with an overline. The rows of the observation vector $\bar{b}_O$ correspond to the following consecutive measurements: $|HH\rangle\langle HH|$, $|HV\rangle\langle HV|$, $|VH\rangle\langle VH|$, $|VV\rangle\langle VV|$, $|HD\rangle\langle HD| - |HA\rangle\langle HA|$, $|HL\rangle\langle HL| - |HR\rangle\langle HR|$, $|DH\rangle\langle DH| - |AH\rangle\langle AH|$, $|LH\rangle\langle LH| - |RH\rangle\langle RH|$, $|VD\rangle\langle VD| - |VA\rangle\langle VA|$, $|VL\rangle\langle VL| - |VR\rangle\langle VR|$, $|DV\rangle\langle DV| - |AV\rangle\langle AV|$, $|LV\rangle\langle LV| - |RV\rangle\langle RV|$, $|\Psi^+\rangle\langle\Psi^+| - |\Psi^-\rangle\langle\Psi^-|$, $|\bar{\Psi}^+\rangle\langle\bar{\Psi}^+| - |\bar{\Psi}^-\rangle\langle\bar{\Psi}^-|$, $|\Phi^+\rangle\langle\Phi^+| - |\Phi^-\rangle\langle\Phi^-|$, $|\bar{\Phi}^+\rangle\langle\bar{\Phi}^+| - |\bar{\Phi}^-\rangle\langle\bar{\Phi}^-|$, where $|\bar{\Phi}^\pm\rangle = (|HH\rangle \pm i|VV\rangle)/\sqrt{2}$ and $|\bar{\Psi}^\pm\rangle = (|HV\rangle \pm i|VH\rangle)/\sqrt{2}$.

E. Pauli matrices based tomography

$$
\bar{b}_P = \begin{bmatrix}
\overline{1277} & 1264 & 132 & \overline{57} & 133 & 1144 & 40 & \overline{113} & \overline{1037} & 95 & \overline{52} & 170 & 55 & 175 & \overline{212} & \overline{1244} & \overline{1102} \\
268 & \overline{197} & \overline{1241} & \overline{1129} & \overline{1115} & \overline{126} & 134 & 1222 & \overline{68} & 1100 & 1032 & 984 & \overline{986} & \overline{825} & 577 & \overline{63} & \overline{59} \\
\overline{82} & \overline{2} & 357 & 196 & 80 & 269 & 98 & 95 & 9 & 71 & \overline{81} & \overline{114} & 102 & \overline{128} & 17 & 33 & 38 \\
107 & 107 & 105 & 39 & 105 & 117 & \overline{79} & 69 & \overline{21} & \overline{58} & \overline{24} & \overline{83} & \overline{41} & \overline{830} & \overline{560} & \overline{49} & \overline{57} \\
143 & \overline{79} & \overline{1222} & \overline{216} & 1107 & \overline{46} & \overline{132} & 1182 & 242 & \overline{1070} & 75 & \overline{65} & \overline{96} & 243 & \overline{23} & 268 & 244 \\
1247 & \overline{1264} & 146 & 264 & 57 & 1167 & 74 & \overline{127} & \overline{940} & 137 & \overline{74} & 23 & \overline{13} & \overline{759} & 248 & \overline{1145} & \overline{1002} \\
\overline{98} & 138 & 225 & 1148 & \overline{269} & \overline{276} & \overline{214} & 224 & \overline{172} & \overline{26} & \overline{1005} & 1049 & \overline{932} & \overline{19} & \overline{120} & \overline{359} & \overline{407} \\
\overline{134} & \overline{68} & \overline{94} & \overline{55} & \overline{87} & \overline{155} & 199 & \overline{61} & 76 & 15 & 27 & \overline{10} & 20 & \overline{745} & \overline{263} & 140 & 138 \\
\overline{45} & 22 & 196 & 1186 & \overline{224} & 245 & 121 & \overline{155} & \overline{34} & \overline{14} & 1054 & \overline{1039} & \overline{973} & \overline{151} & 576 & \overline{93} & \overline{104} \\
137 & \overline{6} & 269 & \overline{177} & \overline{60} & \overline{249} & 90 & \overline{113} & 154 & 148 & \overline{32} & 280 & 33 & 977 & \overline{1342} & 413 & 397 \\
1319 & 1305 & 1273 & 310 & \overline{1150} & \overline{1199} & \overline{970} & 1284 & \overline{1100} & \overline{1130} & 13 & \overline{27} & 55 & \overline{34} & 255 & \overline{1302} & \overline{1316} \\
\overline{19} & \overline{39} & 118 & 90 & 72 & \overline{35} & 976 & 183 & 174 & 44 & \overline{20} & \overline{16} & \overline{10} & 926 & 1487 & 415 & 724 \\
\overline{64} & 82 & 248 & 140 & \overline{249} & 19 & 102 & \overline{176} & \overline{65} & 202 & \overline{16} & \overline{31} & 26 & \overline{440} & 644 & \overline{50} & \overline{45} \\
\overline{292} & 246 & \overline{28} & \overline{33} & \overline{32} & \overline{237} & 79 & 4 & 174 & \overline{41} & 41 & \overline{69} & 29 & 1846 & \overline{1638} & 296 & 370 \\
29 & \overline{47} & 91 & \overline{242} & \overline{16} & 113 & \overline{981} & 158 & \overline{158} & \overline{44} & 188 & \overline{200} & 182 & 54 & 274 & \overline{367} & \overline{680} \\
1397 & 1356 & 1362 & 1290 & 1220 & 1313 & 1000 & 1331 & 1124 & 1157 & 1083 & 1052 & 1004 & 1816 & 1815 & 1367 & 1383
\end{bmatrix}
$$

The negative elements of $\bar{b}_P$ are marked with an overline. The rows of the observation vector $\bar{b}_P$ correspond to the following consecutive measurements: $(|DD\rangle\langle DD| + |AA\rangle\langle AA|) - (|DA\rangle\langle DA| + |AD\rangle\langle AD|)$, $(|DL\rangle\langle DL| + |AR\rangle\langle AR|) - (|DR\rangle\langle DR| + |AL\rangle\langle AL|)$, $(|DH\rangle\langle DH| + |AV\rangle\langle AV|) - (|DV\rangle\langle DV| + |AH\rangle\langle AH|)$, $(|DH\rangle\langle DH| + |AH\rangle\langle AH|) - (|DV\rangle\langle DV| + |AV\rangle\langle AV|)$, $(|LD\rangle\langle LD| + |RA\rangle\langle RA|) - (|LA\rangle\langle LA| + |RD\rangle\langle RD|)$, $(|LL\rangle\langle LL| + |RR\rangle\langle RR|) - (|LR\rangle\langle LR| + |RL\rangle\langle RL|)$, $(|LH\rangle\langle LH| + |RV\rangle\langle RV|) - (|LV\rangle\langle LV| + |RH\rangle\langle RH|)$, $(|LH\rangle\langle LH| + |RH\rangle\langle RH|) - (|LV\rangle\langle LV| + |RV\rangle\langle RV|)$, $(|HD\rangle\langle HD| + |VA\rangle\langle VA|) - (|HA\rangle\langle HA| + |VD\rangle\langle VD|)$, $(|HL\rangle\langle HL| + |VR\rangle\langle VR|) - (|HR\rangle\langle HR| + |VL\rangle\langle VL|)$, $(|HH\rangle\langle HH| + |VV\rangle\langle VV|) - (|HV\rangle\langle HV| + |VH\rangle\langle VH|)$, $(|HH\rangle\langle HH| + |VH\rangle\langle VH|) - (|HV\rangle\langle HV| + |VV\rangle\langle VV|)$, $(|HD\rangle\langle HD| - |HA\rangle\langle HA|) + (|VD\rangle\langle VD| - |VA\rangle\langle VA|)$, $(|HL\rangle\langle HL| - |HR\rangle\langle HR|) + (|VL\rangle\langle VL| - |VR\rangle\langle VR|)$, $(|HH\rangle\langle HH| - |HV\rangle\langle HV|) + (|VH\rangle\langle VH| - |VV\rangle\langle VV|)$, $(|HH\rangle\langle HH| + |HV\rangle\langle HV|) + (|VH\rangle\langle VH| + |VV\rangle\langle VV|)$.

IV. ERROR ANALYSIS

A. Estimated variances

For all the tomographies the vectors of variances for the 17 measured states are given as matrices

$$\sigma^2(b) = \begin{array}{c} \\ b_1 \\ b_2 \\ \vdots \\ b_N \end{array} \begin{array}{c} \begin{array}{cccc} \rho_1 & \rho_2 & \cdots & \rho_{17} \end{array} \\ \begin{bmatrix} \sigma^2(b_{1,1}) & \sigma^2(b_{1,2}) & \dots & \sigma^2(b_{1,17}) \\ \sigma^2(b_{2,1}) & \sigma^2(b_{2,2}) & \dots & \sigma^2(b_{2,17}) \\ \vdots & \vdots & \ddots & \vdots \\ \sigma^2(b_{N,1}) & \sigma^2(b_{N,2}) & \dots & \sigma^2(b_{N,17}) \end{bmatrix} \end{array}.$$

They can be approximated directly with $\sigma^2(b) \approx \sigma^2(\bar{b}) = \bar{b}$ for all the tomographies except the optimal one and the tomography based on the Pauli matrices. For the optimal tomography the matrix of variances reads

$$\sigma^2(b_O) = \begin{bmatrix}
2727 & 2575 & 2844 & 1448 & 127 & 193 & 25 & 2955 & 40 & 27 & 1264 & 809 & 1231 & 2762 & 3831 & 113 & 112 \\
30 & 58 & 116 & 1312 & 2457 & 2364 & 3928 & 72 & 2555 & 2375 & 863 & 1263 & 757 & 2722 & 2773 & 3452 & 4102 \\
126 & 43 & 61 & 648 & 2282 & 2660 & 13 & 23 & 1892 & 2199 & 1277 & 895 & 1141 & 977 & 347 & 1887 & 1295 \\
2706 & 2747 & 2426 & 1751 & 14 & 36 & 35 & 2274 & 8 & 26 & 928 & 1240 & 888 & 802 & 310 & 17 & 23 \\
1352 & 1297 & 1445 & 1362 & 1299 & 1219 & 1936 & 1427 & 1250 & 1198 & 1088 & 1109 & 997 & 2842 & 3309 & 1900 & 2161 \\
1425 & 1456 & 1624 & 1433 & 1211 & 1207 & 1933 & 1515 & 1104 & 1102 & 1141 & 989 & 995 & 2844 & 3246 & 1700 & 2016 \\
1291 & 1199 & 1375 & 1015 & 1199 & 1395 & 23 & 1294 & 964 & 1158 & 1252 & 906 & 1190 & 1858 & 2177 & 955 & 669 \\
1460 & 1427 & 1454 & 1184 & 1068 & 1203 & 16 & 1454 & 785 & 990 & 1058 & 1103 & 973 & 1936 & 2374 & 820 & 583 \\
1421 & 1388 & 1196 & 1158 & 1076 & 1321 & 29 & 1154 & 981 & 1184 & 1104 & 1071 & 1035 & 935 & 284 & 930 & 660 \\
1369 & 1359 & 1204 & 1094 & 1225 & 1410 & 22 & 1118 & 998 & 1258 & 1026 & 1104 & 1070 & 900 & 305 & 968 & 691 \\
1419 & 1428 & 1233 & 1436 & 1124 & 1071 & 1770 & 1182 & 1236 & 1194 & 934 & 1236 & 834 & 1787 & 1563 & 1834 & 2080 \\
1295 & 1253 & 1196 & 1360 & 1352 & 1269 & 2108 & 1088 & 1328 & 1307 & 1055 & 1125 & 1037 & 1820 & 1674 & 1979 & 2329 \\
94 & 41 & 98 & 1018 & 2609 & 2596 & 1889 & 60 & 2194 & 2195 & 1209 & 821 & 1075 & 1533 & 1195 & 2565 & 2506 \\
44 & 53 & 146 & 1065 & 2112 & 2196 & 1772 & 86 & 2074 & 2276 & 1031 & 1114 & 862 & 1685 & 1628 & 2274 & 2316 \\
2757 & 2691 & 2431 & 1027 & 127 & 80 & 104 & 2640 & 74 & 27 & 1301 & 1026 & 1262 & 1778 & 2115 & 150 & 144 \\
2806 & 2583 & 2521 & 1249 & 160 & 164 & 105 & 2580 & 55 & 67 & 1183 & 1049 & 1227 & 1737 & 1964 & 306 & 274
\end{bmatrix}.$$

For the Pauli matrices based tomography the matrix of variances reads

$$\sigma^2(b_P) = \begin{bmatrix}
1323 & 1313 & 1259 & 1206 & 1165 & 1201 & 903 & 1240 & 1093 & 1163 & 1069 & 1052 & 1054 & 1927 & 1799 & 1380 & 1377 \\
1326 & 1329 & 1327 & 1185 & 1142 & 1257 & 853 & 1254 & 1022 & 1189 & 1075 & 1036 & 1032 & 1886 & 1877 & 1384 & 1340 \\
1355 & 1314 & 1304 & 1225 & 1162 & 1234 & 897 & 1238 & 1100 & 1176 & 1093 & 1072 & 1012 & 1823 & 1870 & 1395 & 1375 \\
1355 & 1314 & 1304 & 1225 & 1162 & 1234 & 897 & 1238 & 1100 & 1176 & 1093 & 1072 & 1012 & 1823 & 1870 & 1395 & 1375 \\
1392 & 1322 & 1323 & 1232 & 1228 & 1235 & 1089 & 1243 & 1089 & 1126 & 1042 & 1072 & 1016 & 1953 & 1952 & 1406 & 1455 \\
1370 & 1320 & 1322 & 1230 & 1225 & 1225 & 1032 & 1311 & 1051 & 1090 & 1009 & 1062 & 1000 & 1863 & 1965 & 1396 & 1407 \\
1378 & 1341 & 1325 & 1273 & 1210 & 1236 & 1062 & 1271 & 1057 & 1149 & 1056 & 1114 & 1005 & 1878 & 2024 & 1400 & 1456 \\
1378 & 1341 & 1325 & 1273 & 1210 & 1236 & 1062 & 1271 & 1057 & 1149 & 1056 & 1114 & 1005 & 1878 & 2024 & 1400 & 1456 \\
1387 & 1343 & 1321 & 1261 & 1188 & 1270 & 983 & 1291 & 1116 & 1191 & 1097 & 1090 & 1016 & 1889 & 1797 & 1416 & 1411 \\
1397 & 1408 & 1414 & 1264 & 1218 & 1309 & 978 & 1317 & 1051 & 1180 & 1084 & 1047 & 1033 & 1872 & 1776 & 1334 & 1354 \\
1397 & 1356 & 1362 & 1290 & 1220 & 1313 & 1000 & 1331 & 1124 & 1157 & 1083 & 1052 & 1004 & 1816 & 1815 & 1367 & 1383 \\
1397 & 1356 & 1362 & 1290 & 1220 & 1313 & 1000 & 1331 & 1124 & 1157 & 1083 & 1052 & 1004 & 1816 & 1815 & 1367 & 1383 \\
1387 & 1343 & 1321 & 1261 & 1188 & 1270 & 983 & 1291 & 1116 & 1191 & 1097 & 1090 & 1016 & 1889 & 1797 & 1416 & 1411 \\
1397 & 1408 & 1414 & 1264 & 1218 & 1309 & 978 & 1317 & 1051 & 1180 & 1084 & 1047 & 1033 & 1872 & 1776 & 1334 & 1354 \\
1397 & 1356 & 1362 & 1290 & 1220 & 1313 & 1000 & 1331 & 1124 & 1157 & 1083 & 1052 & 1004 & 1816 & 1815 & 1367 & 1383 \\
1397 & 1356 & 1362 & 1290 & 1220 & 1313 & 1000 & 1331 & 1124 & 1157 & 1083 & 1052 & 1004 & 1816 & 1815 & 1367 & 1383
\end{bmatrix}.$$

B. Estimated error radii

For each tomography and reconstructed state we have estimated the maximum error R as described in the main text. Our results are summarized in the following matrix:

$R =$

	Optimal	MUB	Standard	Pauli	JKMW
ρ_1	0.0786	0.1180	0.1746	0.1926	0.4570
ρ_2	0.0798	0.1243	0.1777	0.1958	0.4286
ρ_3	0.0790	0.1205	0.1785	0.1938	0.4238
ρ_4	0.0798	0.1182	0.1704	0.1843	0.4035
ρ_5	0.0841	0.1298	0.1875	0.2053	0.4614
ρ_6	0.0799	0.1271	0.1833	0.1959	0.4442
ρ_7	0.0922	0.1647	0.2110	0.2578	0.4852
ρ_8	0.0799	0.1264	0.1839	0.1964	0.4680
ρ_9	0.0866	0.1370	0.2006	0.2146	0.4908
ρ_{10}	0.0866	0.1306	0.1910	0.2134	0.4646
ρ_{11}	0.0904	0.1221	0.1825	0.2009	0.4663
ρ_{12}	0.0908	0.1278	0.1834	0.2050	0.4807
ρ_{13}	0.0943	0.1256	0.1854	0.2105	0.4680
ρ_{14}	0.0689	0.0992	0.1207	0.1635	0.3126
ρ_{15}	0.0691	0.1025	0.1270	0.1702	0.3530
ρ_{16}	0.0795	0.1218	0.1737	0.1983	0.4353
ρ_{17}	0.0788	0.1250	0.1734	0.2025	0.4183

Note that the values are multiplied by a factor of 1.04 to compensate for underestimation of $\|\sigma\|$. Standard error is simply given by $r = R/2$.

It follows from the definition of R that $R \propto 1/\sqrt{t}$ is inverse proportional to square root of time t spent on a single tomographic measurement when all the photon counts are performed in equal 5 s time intervals. We would like to compare the tomographic protocols of various numbers of measurement setup settings N and fixed total amount of time spent on all the measurements. To do this with our measured data we postprocess the raw radii $R \to R_{\|} = sR$ by rescaling them by factors of $s = \sqrt{g}$, where $g = N/N_0$ and N_0 is the lowest number of settings for the fastest the protocol.

Using a setup with four bucket detectors allows to gather more precise data in the same time due to measurement paralellization. In this case the scaling factor reads $s = 3/\sqrt{7}$ for all the tomographies (all measurements can be taken using $N = 9$ settings) except the MUB tomography, where $s = 1$ ($N = N_0 = 7$ settings are enough). This means that in the four-detector regime the MUB tomography is 9/7 faster than others allowing gathering more precise data in shorter time. In order to incorporate this fact into our analysis we have multiplied the error radii for all tomographies except the MUB ($s = 1$) by a factor of $s = 3/\sqrt{7} \approx 1.134$, i.e.,

$R_{\|} =$

	Optimal	MUB	Standard	Pauli	JKMW
ρ_1	0.0892	0.1180	0.1980	0.2183	0.5182
ρ_2	0.0904	0.1243	0.2014	0.2221	0.4859
ρ_3	0.0895	0.1205	0.2024	0.2197	0.4805
ρ_4	0.0904	0.1182	0.1932	0.2090	0.4575
ρ_5	0.0954	0.1298	0.2126	0.2328	0.5231
ρ_6	0.0906	0.1271	0.2078	0.2222	0.5038
ρ_7	0.1045	0.1647	0.2392	0.2922	0.5502
ρ_8	0.0906	0.1264	0.2086	0.2227	0.5306
ρ_9	0.0982	0.1370	0.2274	0.2433	0.5565
ρ_{10}	0.0982	0.1306	0.2166	0.2420	0.5268
ρ_{11}	0.1025	0.1221	0.2069	0.2278	0.5287
ρ_{12}	0.1030	0.1278	0.2080	0.2325	0.5451
ρ_{13}	0.1070	0.1256	0.2102	0.2386	0.5306
ρ_{14}	0.0781	0.0992	0.1369	0.1854	0.3544
ρ_{15}	0.0784	0.1025	0.1439	0.1930	0.4002
ρ_{16}	0.0902	0.1218	0.1970	0.2249	0.4936
ρ_{17}	0.0894	0.1250	0.1966	0.2296	0.4743

but this does not change the fact that the smallest error is associated with the optimal tomography.

If there is no paralellization because only two detectors are used ($N_0 = 16$), we obtain $s = 1$ for all the tomographies except the MUB ($s = \sqrt{20}/4$) and standard tomography ($s = 3/2$). Thus, the two tomographies yield larger errors in comparison to the other tomographies due to the equal total time constraint. This is expected, because shorter time per measurement implies larger error per measurement. Thus, in this regime the optimal tomography is even better that it follows directly from our experimental data (estimated errors R).

C. Relative trace distances between the reconstructed states

In order to compare the quality of the matrices reconstructed with different tomographic protocols, we have also calculated the relative trace distances for the respective states in each protocols. Here we omitted the JKMW protocol, because it provides the largest error radius. Having three relative distances (for the remaining three protocols) it is possible to visualize the relative distances between the matrices and their error radii on a plane.

$$T = \begin{array}{c} \\ \rho_1 \\ \rho_2 \\ \rho_3 \\ \rho_4 \\ \rho_5 \\ \rho_6 \\ \rho_7 \\ \rho_8 \\ \rho_9 \\ \rho_{10} \\ \rho_{11} \\ \rho_{12} \\ \rho_{13} \\ \rho_{14} \\ \rho_{15} \\ \rho_{16} \\ \rho_{17} \end{array} \begin{array}{c} \begin{array}{ccc} T(\rho_O,\rho_M) & T(\rho_O,\rho_S) & T(\rho_M,\rho_S) \end{array} \\ \begin{bmatrix} 0.1415 & 0.1004 & 0.1203 \\ 0.1462 & 0.0798 & 0.1048 \\ 0.1018 & 0.1130 & 0.1362 \\ 0.1295 & 0.1786 & 0.1967 \\ 0.0818 & 0.1684 & 0.1924 \\ 0.1234 & 0.1176 & 0.0818 \\ 0.0806 & 0.0483 & 0.0990 \\ 0.1155 & 0.1150 & 0.1541 \\ 0.1613 & 0.1245 & 0.0998 \\ 0.1397 & 0.0956 & 0.1404 \\ 0.0590 & 0.0515 & 0.0591 \\ 0.1000 & 0.1074 & 0.0960 \\ 0.0896 & 0.1000 & 0.0998 \\ 0.0688 & 0.0425 & 0.0686 \\ 0.0718 & 0.0790 & 0.0813 \\ 0.1506 & 0.1270 & 0.1311 \\ 0.1576 & 0.1278 & 0.1179 \end{bmatrix} \end{array}$$

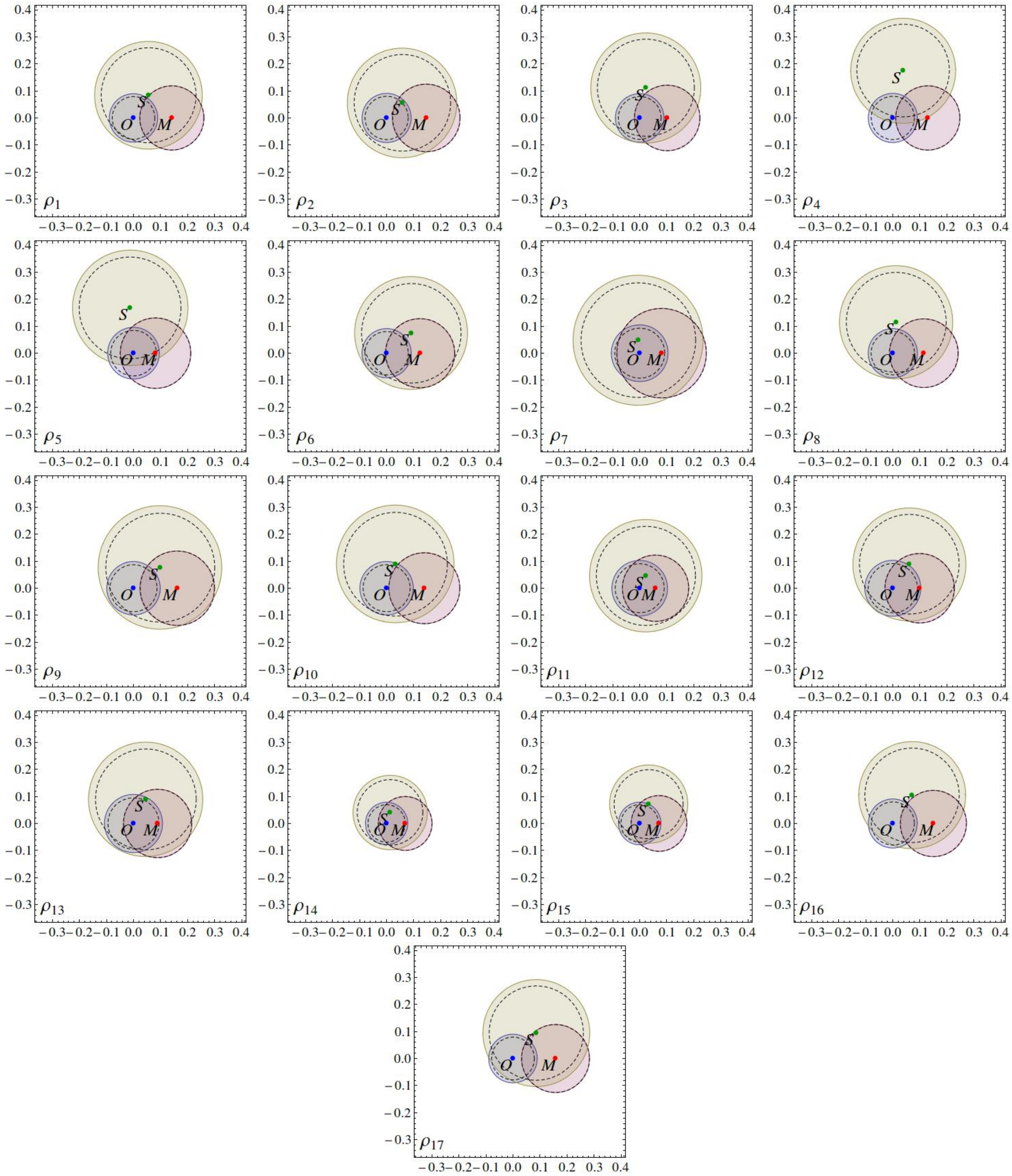

FIG. 4. Relative distances between points representing the reconstructed density matrices and their corresponding circles of the maximum errors $R_{\|}$ optimal (O), standard (S), MUB-based (M) tomographies. The dashed circles correspond to the raw error radii R. The 12 states ρ_n are given in the units of trace distance. The states can be approximated by using Eq. (1) with $\rho_n = |\psi_n\rangle\langle\psi_n|$. The absolute positions of the three points are irrelevant. All the states for $n = 1, ..., 12$ are fully entangled except ρ_7. The states of for $n = 13, ..., 17$ are partially entangled or separable ($n = 13,\ 14$). The ideally reconstructed state lies in the intersection of the error circles of radius $R_{\|}$ (or R).
